# Evidence supporting the existence of a NUPR1-like family of helix-loop-helix chromatin proteins related to, yet distinct from, AT hook-containing HMG proteins

**DOI:** 10.1007/s00894-014-2357-7

**Published:** 2014-07-24

**Authors:** Raul Urrutia, Gabriel Velez, Marisa Lin, Gwen Lomberk, Jose Luis Neira, Juan Iovanna

**Affiliations:** 1 Laboratory of Epigenetics and Chromatin Dynamics, Departments of Biochemistry and Molecular Biology, Biophysics, and Medicine, Epigenomics Translational Program, Center for Individualized Medicine., Mayo Clinic, 200 First Street SW, Guggenheim 10, Rochester, MN 55905 USA; 2 Cell and Molecular Biology Institute, Miguel Hernández University, Elche, Alicante, Spain; 3 Biocomputation and Physics of Complex Systems, Zaragoza, Spain; 4 Centre de Recherché en Cancérologie de Marseille (CRCM), INSERM UMR 1068, CNRS UMR 7258, Aix-Marseille Université and Institut Paoli-Calmettes, 163 Av de Luminy, Campus de Luminy, 13288 Marseille, France

**Keywords:** DNA-binding proteins, NUPR1, Molecular dynamics, High Mobility Group (HMG)

## Abstract

NUPR1, a small chromatin protein, plays a critical role in cancer development, progression, and resistance to therapy. Here, using a combination of structural bioinformatics and molecular modeling methods, we report several novel findings that enhance our understanding of the biochemical function of this protein. We find that NUPR1 has been conserved throughout evolution, and over time it has undergone duplications and transpositions to form other transcriptional regulators. Using threading, homology-based molecular modeling, molecular mechanics calculations, and molecular dynamics simulations, we generated structural models for four of these proteins: NUPR1a, NUPR1b, NUPR2, and the NUPR-like domain of GTF2-I. Comparative analyses of these models combined with extensive linear motif identification reveal that these four proteins, though similar in their propensities for folding, differ in size, surface changes, and sites amenable for posttranslational modification. Lastly, taking NUPR1a as the paradigm for this family, we built models of a NUPR–DNA complex. Additional structural comparisons revealed that NUPR1 defines a new family of small-groove-binding proteins that share structural features with, yet are distinct from, helix-loop-helix AT-hook-containing HMG proteins. These models and inferences should lead to a better understanding of the function of this group of chromatin proteins, which play a critical role in the development of human malignant diseases.

**Electronic supplementary material:**

The online version of this article (doi:10.1007/s00894-014-2357-7) contains supplementary material, which is available to authorized users.

## Introduction

**Fig. 1:**
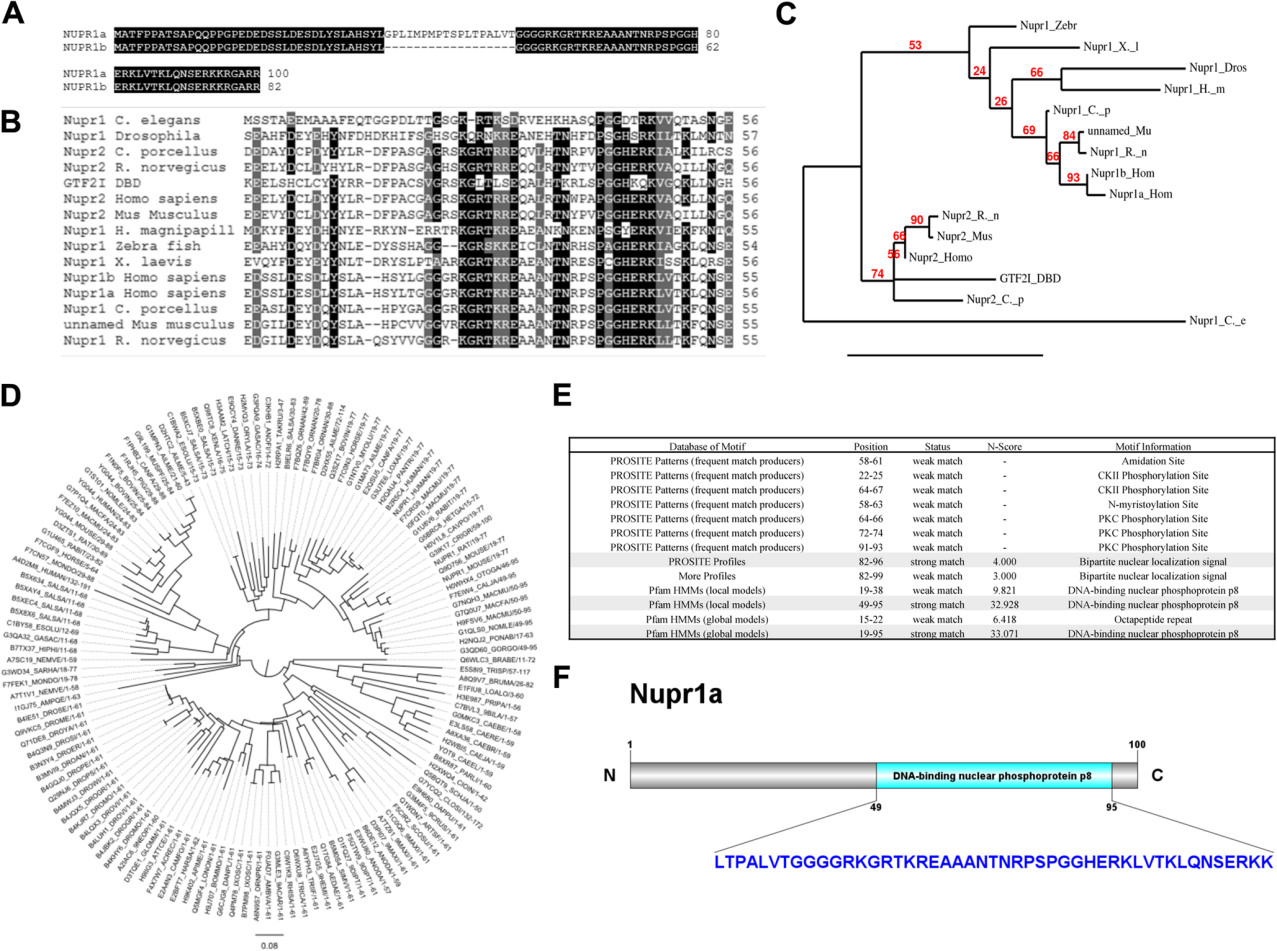
**a**–**f** NUPR1 defines a structurally conserved family of transcriptional regulatory proteins. **a** Pairwise alignment of the two NUPR1 isoforms, highlighting the 18-amino-acid insertion in NUPR1a. **b** Multiple sequence alignment of NUPR1-like and NUPR2-like sequences. Sequences are colored according to percent identity. **c** A neighbor-joining phylogenetic tree was generated from the results of the multiple sequence alignment to display the evolutionary distance between the NUPR1- and NUPR2-like proteins. This representation clearly indicates that NUPR1 and NUPR2 are products of different genes yet share similarities in sequence. **d** A hidden-Markov-model-based domain scan of the NUPR1a sequence yielded 134 individual sequences containing the NUPR1-like DNA-binding domain. These sequences were aligned and used as a seed for further HMM-based domain scans. A phylogenetic tree was constructed to show that the NUPR1-like domain has been conserved across evolution from organisms ranging from nematodes to humans. **e** Domain scan results reveal a DNA-binding nuclear phosphoprotein p8 domain in NUPR1a that has been highly conserved throughout evolution. This domain was predicted by Pfam local and global models to fall within the sequence ranges 49–95 and 19–95, respectively. Additionally, the HMM-based domain scan revealed a conserved bipartite nuclear localization signal located at residues 82–96. This suggests that NUPR1-like proteins have evolved under stringent evolutionary pressures and that their function has been carefully selected. **f** Visualization of the DNA-binding nuclear phosphoprotein p8 domain in relation to the entire NUPR1a sequence

NUPR1, also called p8, is a small nonspecific DNA-binding protein that is induced in response to cell stress stimuli of varying degrees, such as simple culture medium replacement, growth inhibitory signals, starvation, hypoxia, apoptosis inducers, and anticancer drugs [1]. The widely conserved *NUPR1* gene was first discovered after observation of its strong upregulation during the acute-phase response of patients with pancreatitis [2]. Currently unclassified, NUPR1 does not share any significant homology with other proteins. Sequence analyses of NUPR1 reveal that this protein contains a canonical bipartite domain of positively charged amino acids typical of nuclear-localization signals (NLS) [3] and an N-terminal Pro/Glu/Ser/Thr-rich region [4], suggesting nuclear localization and regulation by the ubiquitin/proteasome system. This notion agrees with experimental data indicating that NUPR1 is a short-lived inducible protein which undergoes cytoplasmic-to-nuclear translocation for binding to DNA and regulates gene expression [5]. Interestingly, careful analyses of sequences deposited in protein databases (NCBI and UCSD) show that alternative splicing can produce a longer isoform, named NUPR1a (100 residues), which contains 18 additional amino acids and for which no function has been reported (Fig. 1a). Furthermore, the difference in function and distribution of expression between the two isoforms remains unreported in the literature. Notably, however, all studies performed to date on the biochemistry, biology, and pathobiology of NUPR1 have been performed with the b isoform (82 residues). In this regard, previous characterizations have revealed that NUPR1b exhibits modest primary structural similarity (less than 35 % similarity and below 7 % identity) to the HMG-I/Y class of transcriptional regulators, yet they are very similar in their biochemical properties, including their molecular masses, isoelectric points, hydrophobicity plots, heat stabilities, and charge distributions [6]. In fact, like HMG-I/Y, NUPR1 binds to DNA in vitro [3] and regulates gene expression networks in vivo [7–9]. Nuclear magnetic resonance and circular dichroism analyses using NUPR1 purified from *E. coli* expression systems suggest that this protein may not readily assume a stable secondary structure, and that its tertiary structure is very unstable [5, 6, 10]. These properties have made the traditional structural elucidation of this protein difficult. However, in vitro phosphorylation of a single S residue within NUPR1 increases the propensity of this protein to fold, as well as its ability to bind to DNA [10]. These data, together with the fact that the active form of NUPR1 for the regulation of gene expression requires interaction with other proteins and DNA, suggest that both posttranslational modification and binding to other molecules stabilize the folding of NUPR1 in a manner that modulates its function. However, structural models of NUPR1–DNA and NUPR1–partner protein complexes that can be further used for protein–protein and protein–DNA docking studies, pharmacophore identification, and drug screening have not been developed. In addition to its role in cellular stress, NUPR1 is overexpressed in several types of human cancers, namely in the late stages and metastasis of pancreatic cancer, which is relevant to the fact that pancreatic ductal adenocarcinoma displays outstanding resistance to cell stress. It has also been postulated that NUPR1 also plays a role in the suppression of other tumors in the prostate and the brain [11]. Thus, the functions of NUPR1 appear to be wide-ranging and largely dependent on the context of its expression, signaling-induced posttranslational modifications, and intermolecular interactions. These data guided the efforts expended in the study reported in the present paper, which provides structural models for several members of the NUPR1 family of proteins. Our data derive from detailed molecular analyses of several NUPR1-like proteins, and show that these proteins are a new family of small chromatin regulators that share properties but are still distinct from AT hook-containing HMG family members. The modeling and analyses of molecular properties described here reveals the mechanisms by which NUPR1-like proteins work at atomic resolution, which should be taken into consideration when designing small drug inhibitors of them. Thus, because of the emerging role of members of this family in cancer-associated processes, our data are not only of biochemical but also biomedical relevance.

## Materials and methods

### Primary structure analysis

Sequences similar to NUPR1 were obtained using PSI-BLAST with the BLOSUM80 algorithm in the NCBI database [12]. The obtained sequences were then compared using a flexible multiple sequence alignment program, and some corrections were made by hand to remove gaps in the alignment (Fig. 1b). Multiple sequence alignment was performed using the flexible alignment software MUSCLE [13]. Results from the sequence alignment were then used to generate a phylogenetic tree displaying the interspecies comparison and evolutionary distances (Fig. 1c). Phylogenetic trees (Fig. 1c) were generated using the neighbor-joining method with the BLOSUM62 algorithm [14]. Maximal likelihood analysis was performed using bootstrap analysis (100 replicates) in PHYML 3.0 [15]. Further primary structure analyses of these proteins involved the use of several bioinformatics algorithms for defining linear motifs, such as hidden Markov model (HMM)-based domain scan analyses using the NUPR1 sequences as a seed to search profile databases in the HMMER software package [16], including PeroxiBase profiles, HAMAP profiles, PROSITE patterns, More profiles, Pfam HMMs (local models), Pfam HMMs (global models), PROSITE patterns (frequent match producers), and PROSITE profiles. These profile hidden Markov models use a position-specific scoring system suitable for searching databases for remotely homologous sequences [11].

### Molecular modeling

Using the threading and ab initio modeling algorithms MUSTER [17], I-TASSER [18], QUARK [19], Chunk-TASSER [20], and Pro-sp3-TASSER [21], several potential models of NUPR1a were generated with the primary sequence as input. The best model was then determined through pair-wise model comparisons and statistical analysis of the RMSDs and Z-scores. RMSD and Z-score values were calculated in the PDB Structural Alignment Tool [22] according to the methods described in [23]. Briefly, the Z-score represents the statistical significance of the longest structural alignment path and is calculated by evaluating the probability of finding an alignment path of the same length with the same (or a smaller) number of gaps and distance from a random comparison of structures using a nonredundant set. This relationship is represented by the following equation: *ρ*(0_*j*_1, − *z*) = *ρ*(*D*_*i*_^av^, *D*_*i*_^sd^, *D* ^obs^) ⋅ *ρ*(*G*_*i*_^av^, *G*_*i*_^sd^, *G* ^obs^). The RMSD value represents the difference between two superimposed structures based on their Cα positions. The structures are optimally superimposed as rigid bodies using least-square minimization according to [24]. Furthermore, each model comparison was individually evaluated through qualitative observations, images of the alignments, linear diagrams, and dot plots. As a negative control, each generated model was also compared to a protein with an all-β-sheet structure and an amino acid sequence with no homology to NUPR1 (Phf19, PDB code: 4BD3). Homology modeling was performed using MODELLER [25]. Comparisons of the generated homology models were performed using VADAR version 1.8 [26] and Dali [27].

### Modeling of NUPR–DNA complexes

The three-dimensional complex structure of NUPR1a bound with B-DNA was generated by docking the NUPR1a model into the minor groove of DNA to achieve maximal intermolecular interactions between the two partners using DP-Dock [28]. Intermolecular interactions of the NUPR1–DNA complex, including salt bridge interactions, hydrogen bonds, electrostatic interactions, and hydrophobic interactions, were calculated in the Receptor-Ligand function of Discovery Studio Client 4.0 using the default parameters [29].

### Linear motif analysis

Linear motifs that account for NUPR1’s translocation were identified using the programs PsortII [30] and NetNES [31]. To identify the residues involved in the binding of DNA by NUPR1, we performed calculations using the DP-Bind [32] and DP-Dock [28] algorithms. Prediction of posttranslational modification sites on NUPR1a was performed by compiling and statistically scoring linear motifs for phosphorylation, acetylation, methylation, ubiquitination, and sumoylation as predicted by 30 different software. The software used to predict phosphorylation were NetPhosk 1.0 and 2.0 [33], Kinasephos 2.0 [34], DIPHOS [35], PhosphoSVM [36], Scansite [37], Musite [38], PPSP [39], and GPS 2.0 [40]. Additionally, 3D phosphorylation prediction was performed using Phos3D [41]. Acetylation sites were predicted using PAIL [42], PREDMOD [43], ASEB [44], PLMLA [45], PSKAcePred [46], BRABSB-PHKA [47], LysAcet [48], and EnsemblePail [49]. Methylation sites were predicted using PMeS [50], BPB-PPMS [51], PLMLA [45], and CKSAAP MetSite [52]. Sumoylation sites were predicted using SUMOsp [53], SUMOplot [54], SUMOhydro [55], PCI-SUMO [56], GPS-SBM 1.0 [57], and ELM [58]. Ubiquitination sites were predicted using BDM-PUB [59], CKSAAP UbSite [60], and UbPred [61]. Results from these predictions were then compiled and statistically scored to assign specificity potential to sites that were predicted to undergo modification in NUPR1a. Briefly, for each individual program, we considered sites for which the prediction score was above the cutoff derived using a training set of modified sequences that had been experimentally validated. Subsequently, we developed a meta-prediction score by assigning a maximum score of 1 to sites that were predicted by all of the programs cited. Scores for other programs were numerically expressed relative to this maximum score.

### Molecular dynamics simulations

To evaluate the statistical probability of NUPR1 adopting helical structures versus disordered conformations, we used PrDOS [62], DisorderPredict [63], and POODLE [64]. The generated NUPR1a model was refined by a 60-ns (1-fs time step) molecular dynamics (MD) simulation. The MD simulation of NUPR1a was performed using the all-atom force-field in CHARMm c36b2 at a temperature of 300 K (NVT ensemble) [65]. The molecule was first energy minimized using a two-step protocol of steepest descent and conjugated gradients. All these steps were done using the SHAKE [66] procedure. A distance-dependent dielectrics implicit solvent model was used with a dielectric constant of 80 and a pH of 7.4. Using the same procedure, additional MD simulations were performed on the NUPR1–DNA complex, setting harmonic constraints for the DNA molecule. A total of 120 conformations were sampled from each simulation for further analyses. Briefly, pairwise alignments for each conformation were performed and RMSD values were reported for each comparison. Next, to analyze structural fluctuations across the simulation time, we sampled six models and aligned them to calculate RMSD values at the residue level.

## Results

### NUPR1 defines a structurally conserved family of transcriptional regulatory proteins

The human NUPR1 gene gives rise to two proteins: NUPR1a, which is 100 amino acids long, and NUPR1b, composed of 82 amino acids. Sequence alignment between these two proteins (Fig. 1a) shows that they differ by an internal deletion of 18 amino acids in NUPR1b. Since previous studies have considered these proteins to be unique, we searched for evidence for the existence of homologs as well as evolutionary duplications and transpositions by performing extensive database searches using PSI-BLAST with the BLOSUM80 algorithm. This BLAST method yielded sequences from several organisms, indicating that NUPR1 has been conserved throughout evolution. A flexible multiple sequence alignment (Fig. 1b) was performed to compare these sequences and assess evolutionary distance (Fig. 1c). These comparisons identified a conserved sequence—what we refer to as the “NUPR1-like domain,” which is the most conserved region of these proteins. This can be used as the primary structure signature that characterizes NUPR1-like proteins. Note that we found that, throughout evolution, there have been proteins which are related to the human NUPR1 but display distinct differences that are revealed by the relatedness of their overall primary structures. Further primary structure analyses of these proteins involved the use of several bioinformatics algorithms for defining linear motifs, such as hidden Markov model (HMM)-based domain scan analyses using the NUPR1 sequences as a seed to search profile databases in the HMMER software package [16], including PeroxiBase profiles, HAMAP profiles, PROSITE patterns, More profiles, Pfam HMMs (local models), Pfam HMMs (global models), PROSITE patterns (frequent match producers), and PROSITE profiles. These profile hidden Markov models use a position-specific scoring system suitable for searching databases for remotely homologous sequences. Note that the sequence profiles from these databases were assembled using amino acid composition/position matrices to allow the detection of homology relationships, which are not commonly identified using pairwise alignments by BLAST-related algorithms (Fig. 1e). The results of these analyses demonstrated that NUPR1 contains a domain that is present in proteins conserved in organisms ranging from nematodes to humans (Fig. 1d). Interestingly, we found that this NUPR1-like domain occurs either alone (as in small NUPR1-related proteins) or in combination with other DNA-binding motifs (as in GTF2I-related proteins). Briefly, we identified at least three highly related proteins in humans: NUPR1a and NUPR1b, which are alternatively spliced products of the same gene located in chromosome 16, and a similar protein, which we called NUPR2. Notably, no previous study has reported the characterization of NUPR2. The presence of NUPR2-like proteins is seen in *Homo sapiens*, *Cavia porcellus*, *Mus musculus*, and *Rattus norvegicus*. Quantitative assessment of the similarity of these proteins within and outside the NUPR1-like DNA binding motif is presented in Table S1 of the “Electronic supplementary material” (ESM). These analyses allowed us to develop a consensus sequence that can be used to identify other members of this family across evolution. Combined, the data from primary structure analyses suggest that NUPR1-related proteins define a new group of DNA-binding proteins. Subsequently, we tried to define whether proteins from this group are related to other transcriptional regulators. In this regard, previous studies had suggested that NUPR1 is related to HMG-I/Y-like proteins, which are intrinsically disordered non-histone chromosomal proteins characterized by the presence of three DNA-binding domains called AT-hooks (DBD) that are known to preferentially bind to the minor groove of short stretches of AT-rich DNA [67]. These AT hooks (DBDs) are formed by a conserved core sequence rich in glycine, arginine, and lysine [67]. The first HMG AT-hook, DBD1, differs from DBD2 and DBD3 by the absence of single proline residues that flank the G/R/K-rich core of this domain. Interestingly, we found that NUPR1 contains a single 10-amino-acid-long AT-hook domain that is similar to the HMGA1 DBD1 but lacks significant homology outside of this region. Combined, the analysis provided here indicates that a NUPR1-like sequence defines distinctly identifiable protein groups, that share only this limited motif.

### Molecular modeling reveals that the tridimensional structure of NUPR1-related proteins is related to, yet distinct from, HMG proteins

**Fig. 2:**
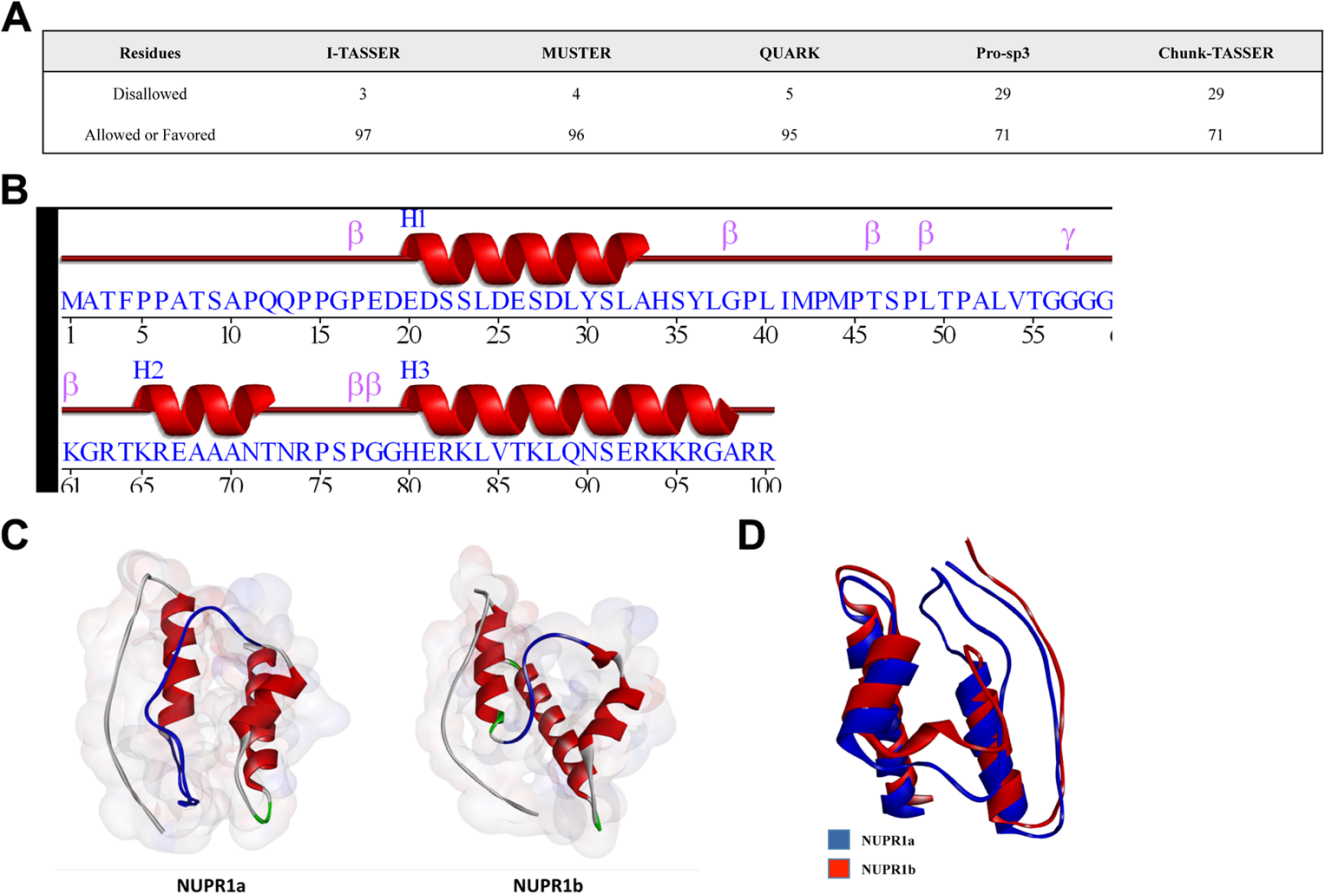
**a**–**d** Comparative modeling of NUPR1a through the combination and scoring of multiple threading algorithms. **a** In addition to our assessment of each threading model, we used PROCHECK [73] to assess their quality. Ramachandran plots of the generated NUPR1a and NUPR1b models revealed that the I-TASSER model had the best overall geometry. With both models having ≥95 % of their residues in favored or allowed regions, I-TASSER outperformed the other threading methods. Pro-sp3 and Chunk-TASSER, both with 29 % of the residues in disallowed regions, were the lowest scoring of the threading algorithms used. **b** A more detailed representation of the secondary structure assignment for the I-TASSER NUPR1a model was generated using PROMOTIF [83]. The protein contains a signature helix-loop-helix motif with 3 distinct helices, 7 β-turns, 2 γ-turns, and 2 helix–helix interactions. Helix 1 contains 14 residues and spans from Glu20 to Ala33. Helix 2 contains 8 residues and spans from Lys65 to Thr72, while helix 3 contains 19 residues and spans from His80 to Ala98. The 7 β-turns are characterized by 4 consecutive nonhelical residues where the α-carbon of the first residue is less than 7 Å from the α-carbon of the fourth residue. The γ-turns of the protein are characterized by 3 consecutive residues with hydrogen bonds between the first and third residues. The psi and phi angles of the second residue fall in the range 75.0° (phi) and –64.0° (psi) associated with a classic γ-turn. **c** Comparison of the two NUPR1 isoforms. **d** Structural alignment of the two NUPR1 isoform models was performed using the Pairwise Structure Alignment Tool in the PDB [22]

**Fig. 3:**
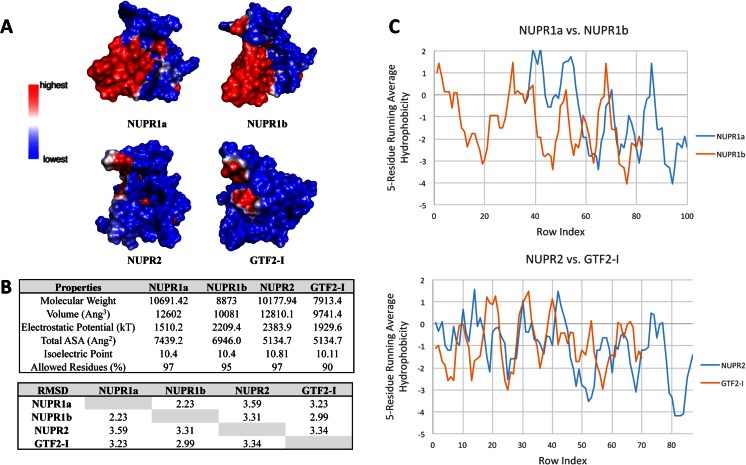
**a**–**c** Comparative molecular properties of members of the NUPR1-like family of proteins: modeling of related proteins was done using the generated NUPR1a model as a template in MODELLER. Here, we developed homology models for NUPR2 and for the DNA-binding domain of the GTF2-I transcription factor. The similarities between NUPR2 and the DNA-binding domain of GTF2-I suggest that the NUPR-like domain has been incorporated into the structure of the transcription factor. **a** Comparison of the surface potentials of the members of the NUPR1-like family of proteins. Although these proteins differ in their total electrostatic potentials, similarities in surface charge distribution can be seen for NUPR1a and NUPR1b and for NUPR2 and GTF2-I. **b** Comparison of structural features of these proteins and RMSD values for their alignments. These proteins have similar isoelectric points but differ in their electrostatic potentials, molecular weights, and volumes. Structural alignments of these models yielded RMSD values of <4, indicating structural consistencies among these proteins. **c** Comparison of the hydrophobicity plots for these proteins indicates that they also differ in this area

We sought to gain insight into the structure and biophysical and biochemical properties of this protein through molecular modeling approaches. We initially attempted to model the structures of NUPR1a and NUPR1b through homology modeling. Unfortunately, however, the level of identity to potential templates deposited in the PDB was below the gold standard of 30 % required for this method [68]. Thus, we resorted to building a model of NUPR1a using multiple algorithms based on threading, ab initio, or mixed approaches and evaluating the consistencies among them. We chose these methods as they have been ranked as among the top systems for protein structure prediction in the CASP7 [69], CASP8 [70], CASP9 [71], and CASP10 [72] experiments. Several potential models of NUPR1a were generated using as input the FASTA file corresponding to the NCBI-deposited primary structure. The software systems used in our studies included MUSTER [17], I-TASSER [18], and QUARK [19], Chunk-TASSER [20], and Pro-sp3-TASSER [21]. Note that all of the models generated revealed that NUPR1a has a propensity to adopt a helix-loop-helix fold, a domain evolutionarily associated with DNA-binding proteins (Fig. S1a of the ESM). Each model comparison was individually evaluated through qualitative observations, images of the alignments, linear diagrams, dot plots, Ramachandran plots, RMSDs (root mean square deviations), and Z-scores [22] (Fig. 2a). As a negative control, each threading model was also compared to a protein with an all-β-sheet structure and an amino acid sequence with no homology to NUPR1 (Phf19, PDB code: 4BD3). RMSDs and Z-scores were used as indicators of model quality since the first measures the average distance in angstroms between superimposed atoms of the two models while the second is a measure of the energy separation between the native fold and misfolds in units of standard deviations of the protein model. Thus, lower RMSD values and higher Z-scores were favored in our analyses. We found that I-TASSER and Quark had the lowest RMSDs and highest Z-scores when compared with the negative control Phf19, as shown in Table 1. Further statistical evaluation of this data was performed by calculating the Pearson’s coefficient (*R* value) of the RMSDs and Z-scores in an all against all models fashion (Table 1). These analyses showed that all *R* values were >0.80, reflecting a strong inverse relationship between RMSDs and Z-scores. However, it is worth noting that worse models (Chunk-TASSER) had higher *R* values than better models did (I-TASSER, Quark). To further estimate model quality, we subsequently generated Ramachandran plots (plot of psi vs. phi angles) using PROCHECK [73]. The I-TASSER model had the best overall geometry, with 97 % of residues in favored and allowed regions. The models generated by Pro-sp3 and Chunk-TASSER both had 29 % of residues in disallowed regions (Fig. 2a). Thus, the latter two models were eliminated due to their poor performance in this area. Combined, these analyses revealed that the model generated by I-TASSER was the best model for representing the folding propensity of NUPR1a. This model was generated using the I-TASSER algorithms, which combine threading approaches and ab initio optimizations, using the templates listed in Fig. S1b of the ESM. The properties of this energy-minimized structure are summarized in Fig. 2b. Briefly, according to this model, several regions of NUPR1a have the tendency to form three α-helices. Helix 1 contains 14 residues and spans from Glu20 to Ala33. Helix 2 contains 8 residues and spans from Lys65 to Thr72, while helix 3 contains 19 residues and spans from His80 to Ala98. Other notable features of this structure include a total volume of 12,602 Å^3^, a total accessible surface area (ASA) of 7439.2 Å^2^, and an electrostatic potential of 1510.2 kT. Using the NUPR1a structure as the template, we developed homology-based models for NUPR1b, NUPR2, and the DNA-binding domain of GTF2-I using MODELLER [25]. Structural comparisons of these proteins were performed based on the RMSDs of their individual alignments, sizes, electrostatics, hydrophobicity plots, and Ramachandran plots. For this approach, we submitted each generated model to the VADAR version 1.8 server [26]. This software analyzes the properties of models generated by homology modeling or traditional structural elucidation techniques by calculating their electrostatic potentials, volumes, accessible surface areas, and hydrogen-bonding interactions. The comparative features among these models are described in Fig. 3. Briefly, these models are of high quality according to their Ramachandran plots (with each model containing ≥90 % of their residues in allowed regions) and their structural alignments (with each comparison yielding RMSD values of <4 Å). Notably, these qualities revealed that these models display appropriate stereochemistry and consistencies among their structures. However, although these models showed similar qualities (stereochemistries), they differed in their volumes, electrostatic potentials, total ASAs, molecular weights, and hydrophobicity plots. The striking structural similarities of the DNA-binding domain of GTF2-I to NUPR2 suggested that a NUPR-like domain has been duplicated and incorporated into this type of larger multi-domain transcriptional regulator. Figure 2c–d displays a structural comparison between NUPR1a and NUPR1b. Note that the 18-amino-acid insertion into NUPR1a takes the form of a flexible loop in the model, which does not compromise any secondary structure. Further analyses of these proteins involved the calculation of intramolecular interactions. For this method, we calculated the hydrophobic interactions, salt bridges, and intramolecular hydrogen bonds in NUPR1a, NUPR1b, NUPR2, and GTF2-I using the Nonbonding Interactions Monitor function in Accelrys Discovery Studio 4.0 [24]. These interactions, which likely contribute to maintaining the structural properties of these proteins in terms of both folding and dynamic conformational changes, are outlined in Table S2a–c of the ESM.

**Table 1:** Scoring of models generated by multiple threading algorithms. Each NUPR1a model generated was aligned with another model using the Pairwise Structure Alignment Tool in the PDB [22]

RMSD	I-TASSER	MUSTER	QUARK	Pro-sp3	Chunk-TASSER	Phf19
I-TASSER	**0**	**4.99**	**4.16**	5.16	**4.76**	8.09
MUSTER	4.99	**0**	**4.64**	6.08	6.35	6.67
QUARK	**4.16**	**4.64**	**0**	**4.71**	6.79	5.17
Pro-sp3	5.16	6.08	4.71	**0**	**4.88**	5.33
Chunk-TASSER	**4.76**	6.35	6.79	**4.88**	**0**	8.37
Phf19	8.09	6.67	5.17	5.33	8.37	0
Z-score	I-TASSER	MUSTER	QUARK	Pro-sp3	Chunk-TASSER	Phf19
I-TASSER	**6.35**	**3.29**	**3.7**	**4.42**	2.3	1.24
MUSTER	3.29	**6.35**	3.07	3.07	1.99	1.99
QUARK	**3.7**	**3.07**	**6.35**	**3.7**	**3.29**	0.73
Pro-sp3	**4.42**	**3.07**	**3.7**	**6.35**	2.3	1.24
Chunk-TASSER	2.3	1.99	3.29	2.3	**6.35**	1.64
Phf19	1.24	1.99	0.73	1.24	1.64	5.6
Standard deviation	I-TASSER	MUSTER	QUARK	Pro-sp3	Chunk-TASSER	
RMSD	**0.38**	0.72	1.03	0.54	0.92	
Z-score	0.92	0.56	**0.47**	0.86	0.61	
R-values	I-TASSER	MUSTER	QUARK	Pro-sp3	Chunk-TASSER	
RMSD v. Z-score	−0.82	**−0.96**	−0.92	−0.85	**−0.86**	
Averages	I-TASSER	MUSTER	QUARK	Pro-sp3	Chunk-TASSER	
RMSD	**4.77**	5.48	**5.15**	5.26	5.59	
Z-score	3.2	2.74	**3.27**	**3.21**	2.63	

RMSD and Z-score values were calculated to evaluate the quality of the model based on the premise that better models would exhibit greater consistencies than other NUPR1a models. Each model was compared against itself as a positive control and against a model of Phf19 (PDB code: 4BD3), a transcriptional repressor, as a negative control. This analysis revealed that the model generated by I-TASSER was the best model for NUPR1a, as it consistently yielded the lowest RMSD values and highest Z-scores for each comparison. The three lowest RMSD values and three highest Z-scores in each column are shown in boldface

**Fig. 4:**
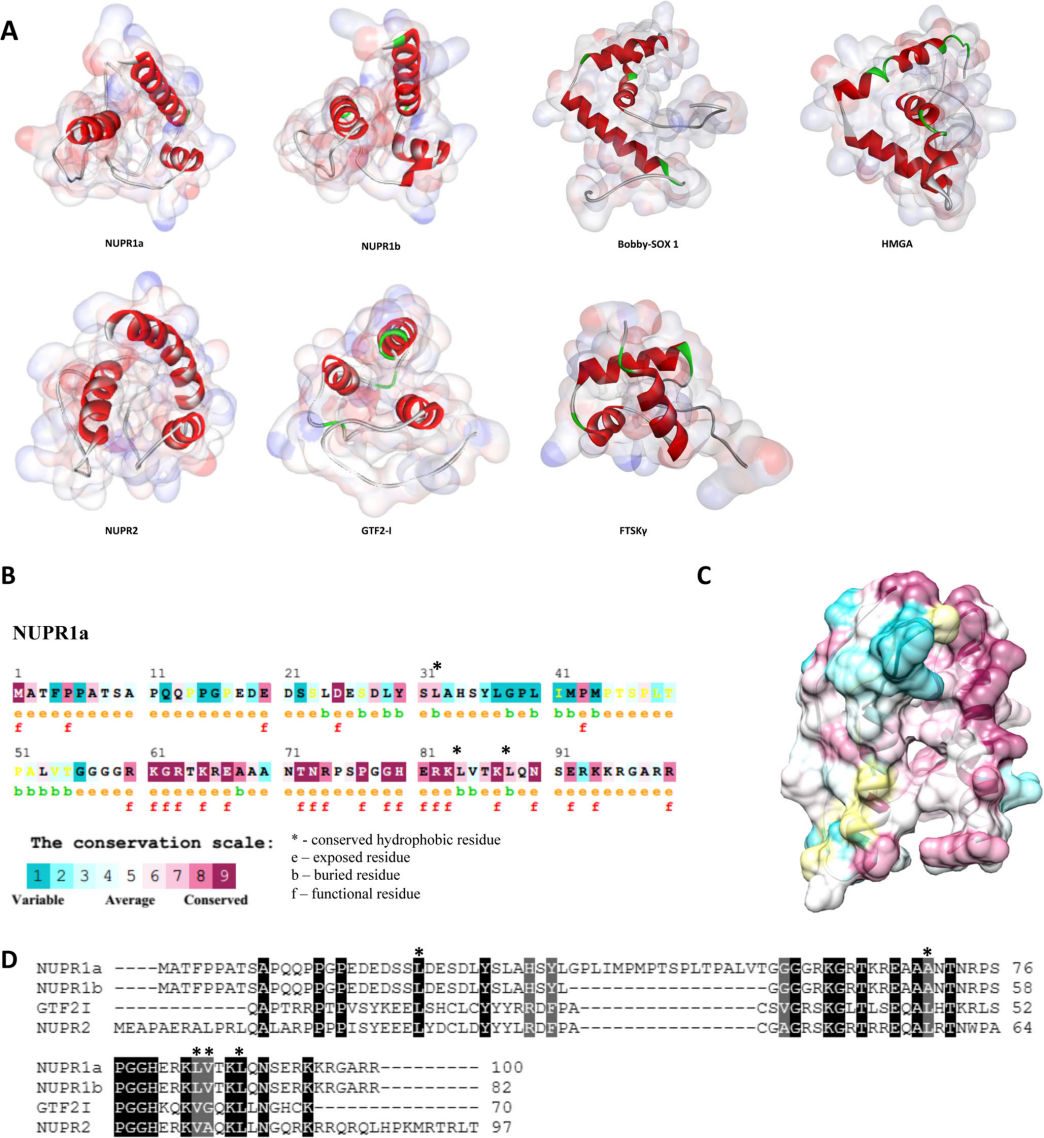
**a**–**d** NUPR1-related proteins are related to, yet distinct from, HMG proteins. **a** A comparison between NUPR1a and previously solved structures of examples of HMG members, such as HMGA (PDB: 2E6O) and Bobby-SOX 1 (PDB: 1WZ6), reveals a clear similarity in tertiary structure. Moreover, we find a striking similarity of these models to the structure of the gamma domain from the bacterial septum-located DNA translocase FtsK (PDB: 2VE8), indicating that NUPR1 is a helix-loop-helix protein which shares similarities with members of the HMG family of chromatin proteins in mammals and preserves the conserved fold seen in some prokaryote transcription factors. **b** Structural conservation of NUPR1a in the context of its primary structure. Residues labeled with a “b” are buried, while residues labeled with an “e” are exposed. Functional residues are indicated by an “f.” **c** Structural conservation within the context of NUPR1’s 3D structure. **d** Multiple sequence alignment reveals the presence of several conserved hydrophobic residues among the human NUPR1 proteins

Comparisons of the models for these members of the NUPR1-like family of proteins with structures that have already been experimentally solved in previous work were made using Dali [27]. The results of this analysis indicated that NUPR1-like proteins possess structural similarities to members of the HMG family of transcription factors. A striking similarity was also detected between these proteins and the gamma domain from the bacterial septum-located DNA translocase FtsK, suggesting that NUPR1-like family members can populate helix-loop-helix conformations, thus preserving the conserved fold that already appears in some prokaryote transcription factors (Fig. 4a). Additionally, we sought to investigate the structural conservation of NUPR1. Briefly, the structure of NUPR1 was evaluated using the ConSurf program for structural conservation [65]. This software identifies functionally important residues in proteins for which there are known three-dimensional structures by estimating their conservation among close sequence homologs. This degree of conservation is then projected onto the three-dimensional structure of the protein in order to visualize regions of the protein that have an important biological function [74]. The results of this analysis are outlined in Fig. 4b–c and reveal conserved amino acids toward the second half of the sequence, suggesting that it is this part of the structure that has been better conserved across evolution. This is an important observation, since it is the second part of the protein that carries important functional domains such as those associated with DNA binding and nuclear localization signals. Furthermore, these results indicate the presence of several conserved hydrophobic amino acids (Leu32, Leu84, and Leu88) that may contribute to the hydrophobic collapse of these proteins (Fig. 4b). In addition to these data, multiple sequence alignment of NUPR1a, NUPR1b, NUPR2, and GTF2-I reveals that hydrophobic residues Leu24 and Leu88 are conserved in 100 % of these proteins, while Ala70 and Val85 are conserved in 50 % of these proteins (Fig. 4d). This structural conservation suggests that these residues may contribute—although not in isolation—to the structural properties of these proteins. Therefore, taken together, our results are consistent with the existence of a family of NUPR1-like proteins which are related to, yet distinct from, AT-hook-containing HMG proteins. Notably, however, the sequence identity between NUPR1 and HMG-I/Y-like proteins is minimal (<10 %).

**Fig. 5:**
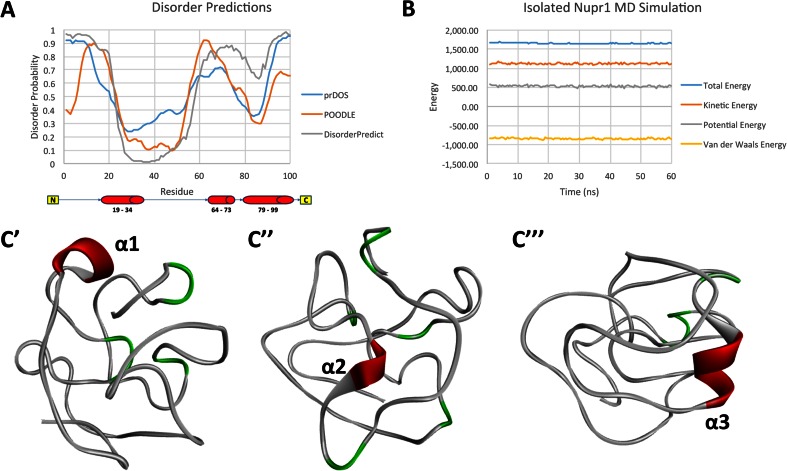
**a**–**c** Analyses of order-to-disorder transitions in NUPR1a, as studied via molecular dynamic simulations. **a** NUPR1 disorder, as determined by statistically based protein disorder prediction algorithms. Residues with disorder probabilities of >0.5 are considered to have a high propensity for disorder. Results from the PrDOS server indicate that helix 1 (residues 19–34) has the lowest probability score for disorder, while helix 2 (residues 64–73) displays the highest probability of disorder. Finally, helix 3 (residues 79–99) displays intermediate propensity for disorder. These results are congruent with the predictions from DisorderPredict and POODLE-L. **b** Energy profile of the isolated NUPR1 MD simulation confirms that the simulation equilibrated. **c** Assembly of NUPR1 conformers observed in a 60-ns MD simulation, showing the conservation of helical folding in *red*. *Green* denotes previous helical structures that underwent a transition to disorder during MD simulation. **c′** NUPR1 conformers with conservation of folding for helix 1. **c″** NUPR1 conformers showing the simultaneous conservation of folding for helix 2. **c′′′** NUPR1 conformers with conservation of folding for helix 3

Interestingly, HMG-I/Y-like proteins show a high tendency to undergo order-to-disorder transitions [75]. This knowledge led us to explore whether NUPR1a also displays a tendency to transition from order to disorder using molecular dynamic simulations combined with protein disorder prediction algorithms and careful consideration of the results from the use of multiple methods used to build the model from Fig. 2. The results obtained using the five threading algorithms indicate that while some of these approaches, namely I-TASSER, QUARK, and Chunk-TASSER, are concordant in the assignment of helical structures to the regions of NUPR1 comprising amino acids 19–34, 64–73, and 79–99, others such as MUSTER and Pro-sp^3^-TASSER identify these areas as randomly coiled (Fig. S1a of the ESM). Since the assignment of secondary structures by this software denotes a statistical probability rather than certainty, we reasoned that these differences reflect a tendency of NUPR1 regions to populate helical and disordered conformations. To further test the validity of this idea, we utilized several approaches that represent the statistical probability that NUPR1 will adopt helical structures versus disordered conformations, including PrDOS [62], DisorderPredict [63], and Prediction of Order and Disorder by Machine Learning [64]. The results of these approaches (shown in Fig. 5a) indicated that the region corresponding to helix 1 (residues 19–34) has the lowest probability scores for disorder. In contrast, the scores were very high for helix 2 (residues 64–73) and intermediate for helix 3 (residues 79–99). Finally, to complement this analysis, we sampled the conformational behavior of NUPR1 over time using MD simulations. Figure 5c shows an assemblage of the different NUPR1 conformations observed during a 60-ns MD simulation. We found that helix 1 remained more frequently folded during the simulation length, helix 3 was present during 15 % of the sampled simulation time, and helix 2 became almost completely disordered. Thus, combined, statistically based disorder prediction methods and MD simulations are congruent with the notion that, like HMG-I/Y-like proteins, NUPR1 displays a significant propensity for disorder.

### Linear motif analyses provide evidence for various mechanisms underlying the functional regulation of NUPR1-like proteins

**Fig. 6:**
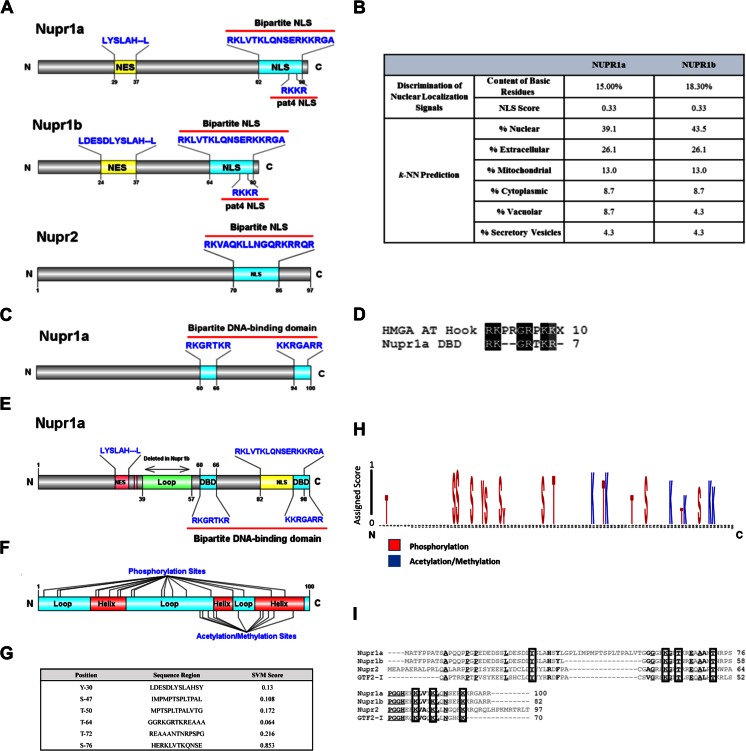
**a**–**i**Assignment of potential posttranslational modification sites within functional domains of NUPR1 by linear motif analyses. **a** Graphical representation of the predicted NES and NLS domains within the context of both NUPR1 isoforms as well as the similarities between them. **b** PsortII prediction of subcellular localization sites for NUPR1a and NUPR1b. PsortII predicted that the NLS signal involves residues 81–96 in NUPR1a and 63–78 for NUPR1b. NLS scores of >0.20 indicate that NUPR1 is a nuclear protein (both isoforms). Results from NetNES predict the nuclear export signal to fall in residues 29–37 for NUPR1a and 24–37 for NUPR1b. The predicted NLS signal follows the typical bipartite pattern of K(K/R)X(K/R) and, likewise, the predicted NES follows the general observed pattern of L_xxx_L_xx_L_x_L. Furthermore, the results of the k-NN prediction indicate a high probability of nuclear localization for both a and b isoforms (39.1 % and 43.5 %, respectively). **c** Graphical representation of the bipartite DNA-binding domain predicted by DP-Bind. **d** Sequence comparison of the predicted DNA-binding domain of NUPR1a and the AT-hook of HMGA. The similarity between these two motifs highlights the reliability of our prediction. **e** Linear motif graph representing the predicted functional linear motifs in NUPR1a. **f** Phosphorylation sites in the context of NUPR1a. Note that residue Thr64 falls within the DNA-binding domain RKGRTKR, suggesting that phosphorylation of this residue could affect the DNA-binding ability of NUPR1. Several predicted phosphorylation sites also fall within the regions of the nuclear export signal and nuclear localization signal. **f** also provides a representation of the predicted acetylation and methylation sites. Several predicted acetylation and methylation sites fall within the DBD and NLS of NUPR1. The results of the predictions suggest that these sites are more likely to be acetylated than methylated. **g** Results of the Phos3D prediction. The generated 3D model of NUPR1a was used as an input for the prediction software. Sites with a positive SVM score are considered to be positive phosphorylation sites based on the spatial context of previously characterized 3D phosphorylation site motifs. **h** Web logo diagram illustrating the specificity potential and assigned scores of the predicted posttranslational modification sites. **i** Multiple sequence alignment of the NUPR1-like proteins reveals differences among them in the positions of potential phosphorylation, acetylation, and methylation sites

Experimental data have demonstrated that NUPR1 undergoes nuclear translocation to access the gene networks that it regulates [5]. Thus, linear motif analyses were performed to identify residues within NUPR1 that account for nuclear localization. We used two bioinformatics methods, PsortII [30] and NetNES [31]. PsortII predicts subcellular localization sites of proteins based on the amino acid sequence using *k *nearest neighbors classifiers (*k-*NN), and NetNES uses a combination of neural networks and hidden Markov models to detect the presence of leucine-rich nuclear export signals. PsortII predicted the NLS signal to be from residue 81 to 96 in NUPR1a and from residues 63–78 for NUPR1b (Fig. 6a). Results from NetNES estimated the nuclear export signal to derive from residues 29–37 for NUPR1a and 24–37 for NUPR1b. The predicted NLS signal followed the typical bipartite pattern of K(K/R)X(K/R) and, likewise, the predicted NES conformed to the general observed pattern of L_xxx_L_xx_L_x_L. These signals should serve as receptor motifs on NUPR1 for importins and exportins to bind. The similarity of the locations of these signals in NUPR1a and b suggest comparable, if not identical, interactions related to these signals for both proteins (Fig. 6a–b). Furthermore, we identified a bipartite nuclear localization signal on NUPR2, suggesting that this protein may have similar functions to NUPR1. However, it should be noted that, in contrast to NUPR1a and NUPR1b, NUPR2 and the NUPR1-like domain of GTFI do not contain a nuclear export signal, indicating that they may differ in how they undergo nuclear-to-cytoplasmic translocation. We used DP-Bind to identify residues involved in DNA binding by NUPR1 [32]. This software implements three machine learning methods—support vector machine (SVM), kernel logistic regression (KLR), and penalized logistic regression (PLR)—to predict DNA-binding and RNA-binding residues from primary structure features, including the side-chain p*K*_a_ value, hydrophobicity index, and molecular mass of an amino acid. Figure 6c provides a graphical representation of the results obtained with this approach, which predicted that the sequences RKGRTKR and KKRGARR form a bipartite DNA-binding domain. Note that the composition of the RKGRTKR sequence expected to interact with nucleic acid bases is similar to the AT-hook DNA-binding motif found in HMG-I/Y-like proteins, highlighting the reliability of this result (Fig. 6d).

Since NUPR1 functions in the regulation of cancer-associated gene expression networks, it is important to gain insight into the mechanisms by which these proteins are either activated or inactivated. Thus, we reasoned that signaling-induced post-translational modifications as well as protein–proteins and protein–DNA interactions may participate in these mechanisms. To determine potential post-translational modification sites, extensive linear motif analysis was performed on the primary structure of NUPR1a using 30 algorithms and prediction software. First, posttranslational modification (such as phosphorylation, acetylation, methylation, sumoylation, and ubiquitination) sites were predicted using NetPhosk 1.0 [33], GPS 2.0 [40], Musite [38], Scansite [37], PREDMOD [43], PLMLA [45], ASEB [44], SUMOsp [53], SUMOplot [54], PCI-SUMO [56], GPS-SBM [57], and ELM [58]—various modification prediction algorithms that produce neural network predictions of modification sites based on a set of previously validated sites. Second, a set of methods utilizing support vector machines (SVM) was used to predict sites, namely Kinasephos 2.0 [34], PhosphoSVM [36], PSKAcePred [46], LysAcet [48], and CKSAAP MetSite [52]. Additionally, DIPHOS [35], PPSP [39], PAIL [42], BRABSB-PHKA [47], EnsemblePail [49], PMeS [50], BPB-PPMS [51], SUMOhydro [55], and CKSAAP UbSite [60] were used to predict modification sites based on machine learning methods such as kernel logistic regression (KLR) and Bayesian decision theory. Results from these predictions were then compiled and statistically scored in order to assign specificity potential to sites that were predicted to undergo modification in NUPR1a. Briefly, for each distinct program, we considered sites for which the prediction score was above the cutoff derived using a training set of modified sequences that had been experimentally validated. Subsequently, we developed a meta-prediction score that assigned a maximum score of 1 to sites that were predicted by all of the programs cited. The scores for the other programs were normalized to a maximum score of 1 (Table S3 in the ESM). Figure 6f shows a graphical representation of these results. Results from the linear motif analysis revealed that phosphorylation could occur throughout the entire sequence of the protein and that potential acetylation/methylation sites are present in the second half of the sequence. Ubiquitination and sumoylation sites were predicted with very low probability and displayed low specificity potential (Table S3d – 3e). Interestingly, several of the predicted modification sites fell within the DNA-binding region, displaying high specificity potential (Fig. 6g). Subsequently, we compared the linear motifs present in NUPR1a with the primary structures of NUPR1b, NUPR2, and GTF2-I. This comparison is highlighted in Fig. 6i. Multiple sequence alignment of these proteins revealed differences in the positions of potential phosphorylation, acetylation, and methylation sites among these proteins. Notably, the loop region of NUPR1a contains posttranslational modification sites that are not present in the other NUPR1-like proteins. While some potential modification sites are found in all NUPR1-like proteins, there are also differences. This suggests that, in addition to differences among them in terms of size and surface charge, these proteins have undergone a degree of functional specialization, potentially enabling them to be differentially regulated by distinct signaling pathways.

### Modeling NUPR1–DNA complexes

**Fig. 7:**
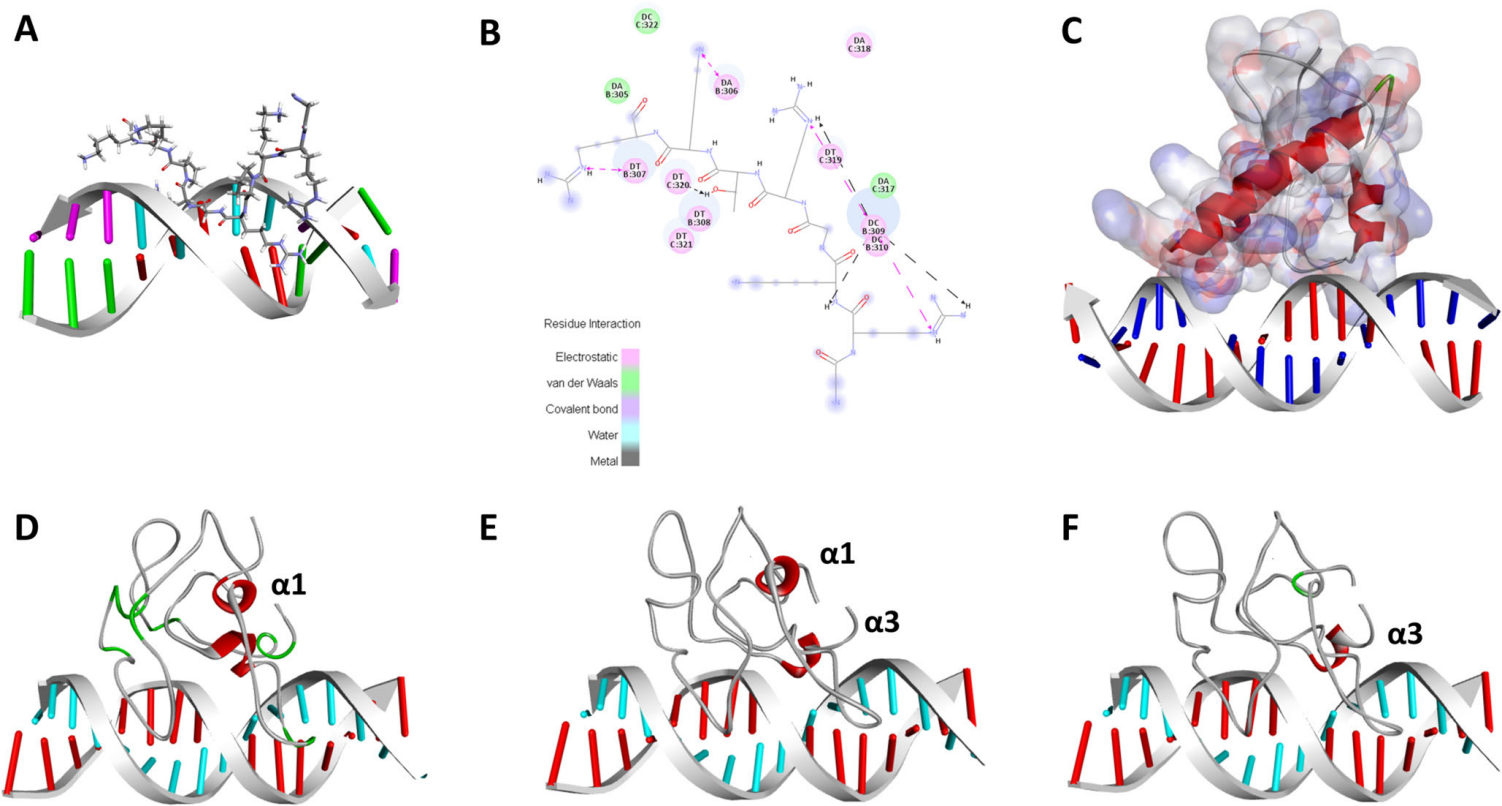
**a**–**f** Modeling NUPR1–DNA complexes. **a** 3D characterization of the NUPR1 DNA-binding domain using a homology-based approach. To achieve this, we used manual docking to superimpose the corresponding region of NUPR1 onto the HMG AT-hook. **b** A simplified view of the bonding interactions between NUPR1 and DNA. **c** 3D model of the NUPR1–DNA complex generated by DP-Dock. Representative models are subsequently selected by clustering and ranking according to their DNA–protein interfacial energies. **d** MD simulations were used to study the order-to-disorder transition of the NUPR1–DNA complex. NUPR1 remains bound to the minor groove of DNA throughout the length of the simulation. Conservation of helical folding is denoted in *red*, while *green* marks previous helical structures that undergo a transition to disorder during MD simulation. The first snapshot shows helix 1. **e** The second snapshot shows helices 1 and 3. Binding to the ideal B-DNA helix stabilizes helix 3, particularly its first half. **f** The third frame shows another view of the helix 3 formed

Our prediction of a DNA-binding domain within the sequence of NUPR1a prompted us to generate a model of NUPR1 bound to DNA. To do this, we applied two well-validated methods. We developed a homology-based model as the first 3D approach to characterize the NUPR1 DNA-binding domain. This model relies upon the previously solved NMR structure of the first hook of HMG-I/Y bound to DNA (PDB: 3UXW). Because of its simplicity, this model lent itself to using manual docking to superimpose the corresponding region of NUPR1 onto the highly homologous HMG-I/Y AT-hook (Fig. 6d). Next, we performed minimization followed by a 2-ns MD simulation. The NUPR1–DNA complex obtained through this homology-based approach is shown in Fig. 7a. This complex was maintained through ionic, van der Waals, and hydrogen-bonding interactions, which are represented graphically in Fig. 7b. The second method, DP-Dock, uses a nonspecific B-DNA to probe the binding site on a 3D model of a protein that is known to bind DNA but for which the specific amino acid to nucleic acid base contacts are unknown. Given the structure of a DNA-binding protein, the method first automatically generates an ensemble of protein–DNA complexes obtained by rigid-body docking with nonspecific canonical B-DNA molecules with the sequence A10–T10 [28]. Models are subsequently selected by clustering and ranking them according to their DNA–protein interfacial energies [28]. Figure 7c shows that this method was successful in generating a NUPR1–DNA complex where the amino acid to base contacts were primarily given by the same RKGRTKR/KKRGARR sequence identified through DP-bind, as shown in Fig. 7c. Analyses of the protein–DNA interphase indicated that residues Arg60, Lys61, and Lys65 occupy the minor groove of DNA, while Arg96, Arg99, and Arg100 further stabilize the complex by binding to the phosphate-rich backbone. The ionic and hydrogen-bonding interactions that define the protein–DNA binding interphase are listed in Tables 2 and 3. In addition, analyses of the DNA-bound NUPR1 complex suggest that this protein prefers to recognize the minor groove of DNA. Notably, these residues have been experimentally shown to be involved in DNA binding [4] since their NMR signals are broadened beyond detection in the presence of DNA, as with the other residues.

**Table 2:** Bonding patterns of the wild-type and mutant NUPR1a–DNA complexes: results of interface analysis performed on the wild-type NUPR1–DNA complex in order to investigate contact residues between NUPR1 and the minor groove of DNA. The complex was subjected to a 2,000-step minimization using steepest descent followed by a 2,000-step conjugated gradient minimization with harmonic restraints on all nucleic acid groups. Contact residues between NUPR1 and DNA were analyzed by defining an interface as a contact area with a maximum salt-bridge distance of 5.0 Å

Receptor residue	Ligand residue	Salt-bridge interaction constituents	Distance (Å)
C:A7	A:Arg60	C:A7:OP2–A:Arg60:NH1	4.77
D:T29	A:Lys61	D:T29:OP2–A:Lys61:NZ	3.65
C:A7	A:Lys65	C:A7:OP2–A:Lys65:NZ	2.53
D:T22	A:Arg96	D:T22:OP2–A:Arg96:NH1	2.61
D:T21	A:Arg99	D:T21:OP2–A:Arg99:NH1	2.56
D:T22	A:Arg100	D:T22:OP2–A:Arg100:NH1	4.96

**Table 3:** Bonding patterns of the wild-type and mutant NUPR1a–DNA complexes: results of an analysis of the hydrogen-bonding interactions between NUPR1 and DNA, which was performed by defining an interface as a contact area with a maximum hydrogen-bond distance of 2.5 Å

Receptor residue	Ligand residue	Interaction constituents	Distance (Å)
C:A8	A:Gly59	A:Gly59:HN–C:A8:O5	2.29
C:A8	A:Arg60	A:Arg60:HN–C:A8:OP1	1.87
C:A7	A:Arg60	A:Arg60:HH12–C:A7:OP1	1.75
C:A7	A:Arg60	A:Arg60:HH21–C:A7:O5	1.63
D:T29	A:Lys61	A:Lys61:HZ2–D:T29:OP1	1.68
D:T29	A:Lys61	A:Lys61:HZ3–D:T29:OP1	2.37
D:T29	A:Lys61	A:Lys61:HZ3–D:T29:O5	1.57
D:T28	A:Arg63	A:Arg63:HH11–D:T28:O2	1.8
D:T27	A:Arg63	A:Arg63:HH12–D:T27:O2	1.97
C:A7	A:Lys65	A:Lys65:HZ2–C:A7:OP1	1.77
C:A7	A:Lys65	A:Lys65:HZ2–C:A7:OP2	2.32
C:A7	A:Lys65	A:Lys65:HZ3–C:A7:OP2	1.75
D:T31	A:Arg66	A:Arg66:HE–D:T31:OP1	2.02
D:T31	A:Arg66	A:Arg66:HH21–D:T31:OP1	1.73
D:T31	A:Arg66	A:Arg66:HH21–D:T31:O5	1.88
D:T22	A:Arg96	A:Arg96:HH12–D:T22:OP1	2.35
D:T22	A:Arg96	A:Arg96:HH12–D:T22:OP2	1.69
D:T22	A:Arg96	A:Arg96:HH22–D:T22:OP2	2.49
D:T22	A:Arg96	A:Arg96:HH22–D:T22:O5	1.62
D:T21	A:Arg99	A:Arg99:HH12–D:T21:OP2	1.69
D:T22	A:Arg100	A:Arg100:HH12–D:T22:OP1	1.73
D:T22	A:Arg100	A:Arg100:HH22–D:T22:OP1	1.75

MD simulations (60 ns) suggest that the interaction between NUPR1 and this B-DNA molecule involves the intermolecular interactions listed in Tables 2, 3, and 4. Thus, combined, the three methods utilized agree in revealing that NUPR1 has the ability to bind to DNA via a bipartite domain composed of an AT-hook-like motif at the N-terminus and a stretch of basic residues at its C-terminus. Subsequently, with the goal of better characterizing the ability of NUPR1 to bind to DNA, we performed in silico mutational analyses in which key residues of interest were changed to either glutamic acid or a residue of the opposite charge and molecular dynamic simulations were implemented. Table 5 shows the NUPR1 residues targeted for study and their corresponding substitutions. Note that these mutations disrupted the bonding pattern observed in the WT NUPR1–DNA complex, which—according to the so-called “additive” model of TF-DNA binding energy [76]—should decrease the strength of these intermolecular interactions. Since all of the algorithms that are widely used for in vivo motif discovery adopt this additive model [77], these data should help to benchmark future ChIP-Seq experiments for genome-wide mapping of NUPR1-binding sites in human, using both the wild-type and mutant forms of this protein. We next studied the order-to-disorder transition of this complex using MD simulations. Interestingly, we observed that—similar to the homology-based model—the HMG-I/Y-like AT-hook motif of NUPR1 remains bound to the minor groove of DNA. We also observed that binding to the ideal B-DNA helix stabilizes helix 3, which persists more frequently upon its formation than helix 1 during the simulation, particularly its first half (Fig. 7d–f). This result suggests that, similar to what has been described for other transcription factors, some regions of NUPR1 show the potential to be stabilized by binding to their partners.

**Table 4:** Bonding patterns of the wild-type and mutant NUPR1a–DNA complexes: electrostatic and hydrophobic interactions between NUPR1 and DNA

Name	Distance	Category	Type	From	From chemistry	To	To chemistry
A:Arg60:NH2–C:A7:O1P	3.14	Electrostatic	Attractive charge	A:Arg60:NH2	Positive	C:A7:O1P	Negative
A:Arg60:NH2–C:A8:O1P	4.75	Electrostatic	Attractive charge	A:Arg60:NH2	Positive	C:A8:O1P	Negative
A:Lys61:NZ–D:T29:O1P	2.38	Electrostatic	Attractive charge	A:Lys61:NZ	Positive	D:T29:O1P	Negative
A:Arg63:NH1–D:T29:O2P	5	Electrostatic	Attractive charge	A:Arg63:NH1	Positive	D:T29:O2P	Negative
A:Lys65:NZ–C:A7:O2P	4.51	Electrostatic	Attractive charge	A:Lys65:NZ	Positive	C:A7:O2P	Negative
A:Lys95:NZ–A:Asp28:OD1	4.94	Electrostatic	Attractive charge	A:Lys95:NZ	Positive	A:Asp28:OD1	Negative
A:Lys95:NZ–A:Glu92:OE2	4.98	Electrostatic	Attractive charge	A:Lys95:NZ	Positive	A:Glu92:OE2	Negative
A:Arg96:NH2–D:T22:O1P	4.56	Electrostatic	Attractive charge	A:Arg96:NH2	Positive	D:T22:O1P	Negative
A:Arg99:NH1–D:T22:O2P	4.75	Electrostatic	Attractive charge	A:Arg99:NH1	Positive	D:T22:O2P	Negative
A:Arg99:NH2–A:Asp21:OD1	5.53	Electrostatic	Attractive charge	A:Arg99:NH2	Positive	A:Asp21:OD1	Negative
C:A6–C:A7	4.11	Hydrophobic	π–π stacked	C:A6	π orbitals	C:A7	π orbitals
C:A6–C:A7	4.41	Hydrophobic	π–π stacked	C:A6	π orbitals	C:A7	π orbitals
C:A6–C:A7	3.57	Hydrophobic	π–π stacked	C:A6	π orbitals	C:A7	π orbitals
C:A7–C:A6	4.14	Hydrophobic	π–π stacked	C:A7	π orbitals	C:A6	π orbitals
C:A7–C:A8	4.11	Hydrophobic	π–π stacked	C:A7	π orbitals	C:A8	π orbitals
C:A7–C:A8	4.41	Hydrophobic	π–π stacked	C:A7	π orbitals	C:A8	π orbitals
C:A7–C:A8	3.57	Hydrophobic	π–π stacked	C:A7	π orbitals	C:A8	π orbitals
C:A8–C:A7	4.14	Hydrophobic	π–π stacked	C:A8	π orbitals	C:A7	π orbitals
C:A8–C:A9	4.11	Hydrophobic	π–π stacked	C:A8	π orbitals	C:A9	π orbitals
C:A8–C:A9	4.41	Hydrophobic	π–π stacked	C:A8	π orbitals	C:A9	π orbitals
C:A8–C:A9	3.57	Hydrophobic	π–π stacked	C:A8	π orbitals	C:A9	π orbitals
C:A9–C:A8	4.14	Hydrophobic	π–π stacked	C:A9	π orbitals	C:A8	π orbitals
D:T20–D:T21	4.07	Hydrophobic	π–π stacked	D:T20	π orbitals	D:T21	π orbitals
D:T21–D:T22	4.07	Hydrophobic	π–π stacked	D:T21	π orbitals	D:T22	π orbitals
D:T22–D:T23	4.07	Hydrophobic	π–π stacked	D:T22	π orbitals	D:T23	π orbitals
D:T23–D:T24	4.07	Hydrophobic	π–π stacked	D:T23	π orbitals	D:T24	π orbitals
D:T27–D:T28	4.07	Hydrophobic	π–π stacked	D:T27	π orbitals	D:T28	π orbitals
D:T28–D:T29	4.07	Hydrophobic	π–π stacked	D:T28	π orbitals	D:T29	π orbitals
D:T29–D:T30	4.07	Hydrophobic	π–π stacked	D:T29	π orbitals	D:T30	π orbitals
D:T30–D:T31	4.07	Hydrophobic	π–π stacked	D:T30	π orbitals	D:T31	π orbitals
A:Ala2–A:Pro45	4.24	Hydrophobic	Alkyl	A:Ala2	Alkyl	A:Pro45	Alkyl
A:Pro5–A:Met44	5.01	Hydrophobic	Alkyl	A:Pro5	Alkyl	A:Met44	Alkyl
A:Ala10–A:Ala52	3.82	Hydrophobic	Alkyl	A:Ala10	Alkyl	A:Ala52	Alkyl
A:Leu32–A:Leu88	4.93	Hydrophobic	Alkyl	A:Leu32	Alkyl	A:Leu88	Alkyl
A:Ala33–A:Leu53	3.24	Hydrophobic	Alkyl	A:Ala33	Alkyl	A:Leu53	Alkyl
A:Leu40–A:Met42	5.15	Hydrophobic	Alkyl	A:Leu40	Alkyl	A:Met42	Alkyl
A:Met42–A:Met44	4.7	Hydrophobic	Alkyl	A:Met42	Alkyl	A:Met44	Alkyl
A:Pro43–A:Met1	4.25	Hydrophobic	Alkyl	A:Pro43	Alkyl	A:Met1	Alkyl
A:Val54–A:Leu29	4.82	Hydrophobic	Alkyl	A:Val54	Alkyl	A:Leu29	Alkyl
A:Ala98–A:Arg100	4.58	Hydrophobic	Alkyl	A:Ala98	Alkyl	A:Arg100	Alkyl
A:Phe4–A:Ala2	4.77	Hydrophobic	π-Alkyl	A:Phe4	π orbitals	A:Ala2	Alkyl
A:Tyr30–A:Ala33	4.69	Hydrophobic	π-Alkyl	A:Tyr30	π orbitals	A:Ala33	Alkyl
A:Tyr30–A:Leu53	4.66	Hydrophobic	π-Alkyl	A:Tyr30	π orbitals	A:Leu53	Alkyl
A:Tyr36–A:Pro5	4.83	Hydrophobic	π-Alkyl	A:Tyr36	π orbitals	A:Pro5	Alkyl
A:His80–A:Pro77	5.05	Hydrophobic	π-Alkyl	A:His80	π orbitals	A:Pro77	Alkyl

**Table 5:** Bonding patterns of the wild-type and mutant NUPR1a–DNA complexes: results of mutational analyses performed to better characterize the ability of NUPR1a to bind to DNA. In these mutational analyses, key residues of interest were changed to either glutamic acid or a residue of the opposite charge and MD simulations were implemented. The mutated NUPR1–DNA complex was subjected to a 2-ns molecular dynamics simulation. The resulting complex no longer contained its original ionic interactions. Instead, salt-bridge interactions were formed at Arg66, Arg93, and Lys95. These changes highlight the functional importance of modifications to the original DNA-binding residues in NUPR1a. Ongoing mutational analysis will lend insight into the posttranslational modifications that either enhance or inhibit its DNA binding

Wild type			
Receptor residue	Ligand residue	Salt-bridge interaction constituents	Distance (Å)
C:A7	A:Arg60	C:A7:OP2–A:Arg60:NH1	4.77
D:T29	A:Lys61	D:T29:OP2–A:Lys61:NZ	3.65
C:A7	A:Lys65	C:A7:OP2–A:Lys65:NZ	2.53
D:T22	A:Arg96	D:T22:OP2–A:Arg96:NH1	2.61
D:T21	A:Arg99	D:T21:OP2–A:Arg99:NH1	2.56
D:T22	A:Arg100	D:T22:OP2–A:Arg100:NH1	4.96
Glutamic acid mutant			
C:A7	A:Arg66	C:A7:OP2–A:Arg66:NH1	3.91
C:A7	A:Arg93	C:A7:OP2–A:Arg93:NH1	3.27
C:A8	A:Arg93	C:A8:OP2 – A:Arg93:NH1	5.35
D:T21	A:Lys95	D:T21:OP2–A:Lys95:NZ	3.08

**Fig. 8:**
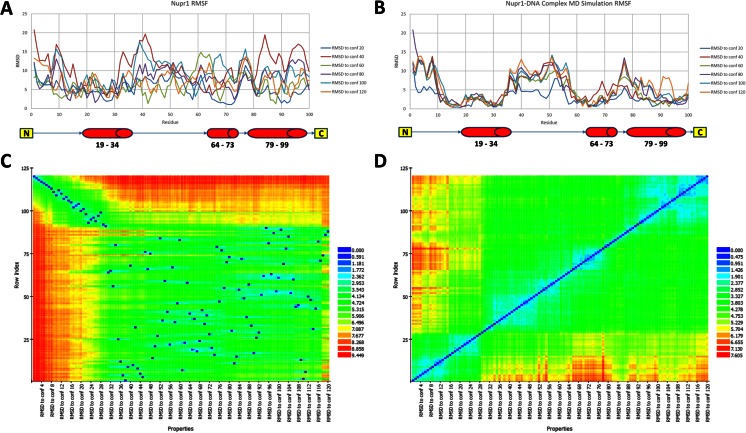
**a**–**d** Binding of NUPR1a to the ideal B-DNA helix provides stabilization of the protein’s helix motifs. **a** Root mean square fluctuation analysis of the isolated NUPR1a MD simulation reveals that the protein is highly disordered across a standard dynamics cascade. These results are congruent with the disorder algorithm predictions which suggested that the residues spanning helix 1 (19–34) are the least disordered (Fig. 5). **b** Root mean square fluctuation analysis of the NUPR1a–DNA complex MD simulation reveal that the residues spanning each α-helix in the complex are the least disordered. **c** A total of 120 conformations were sampled from each simulation for further analyses. Pairwise alignments for each isolated NUPR1 conformation were performed and RMSD values were reported for each comparison. The results of this analysis are represented visually as a heat map to show that the isolated protein undergoes more order-to-disorder transitions. **d** Pairwise alignments for each NUPR1–DNA complex conformation reveal that the complex is more stable across the 60-ns MD simulation. These results further support the hypothesis that the binding of NUPR1a to the ideal B-DNA helix stabilizes the protein

To further gain insight into the stabilizing effects of binding to the ideal B-DNA helix, we performed conformational sampling and analysis of both the isolated NUPR1 MD simulation and that of the NUPR1–DNA complex. Briefly, we sampled six conformations from each simulation and performed structural alignments to calculated RMSD values at the residue level. The results of this root mean square fluctuation (RMSF) analysis reveal that the isolated NUPR1 is disordered; it undergoes wide structural fluctuations in a standard dynamics cascade (Fig. 8a). Alternatively, RMSD analysis of conformations in the MD simulation show that the NUPR1–DNA complex can undergo disorder transitions but is more stable at the residues that span each α-helix (Fig. 8b). To further test this idea, we performed pair-wise structural alignments of each conformation in both simulations and generated heat maps to visualize the results of these alignments (Fig. 8c–d). These results also suggest that NUPR1 undergoes rapid order-to-disorder transitions but can be stabilized in some regions by its binding to DNA.

## Discussion

Here, we report several novel findings that advance our understanding of the biochemical functions of NUPR1, including the first description of a NUPR1-like family of helix-loop-helix proteins which present similarities to helix-loop-helix containing chromatin proteins in mammals and preserve the conserved fold seen in some prokaryotic transcription factors. Our primary structure analyses defined a NUPR1-like domain that has been conserved across evolution from nematodes to humans and diverges to form a similar but uncharacterized protein of a different gene, which we call NUPR2. Interestingly, the conserved NUPR2-like domain is seen in other DNA-binding proteins, such as GTF2-I. These results suggest that the structure and likely the function of the domains formed by NUPR1-like sequences have been carefully maintained throughout evolution. We also report the presence of functionally important linear motifs within NUPR1, such as a leucine-rich nuclear export signal, a signature bipartite nuclear localization signal, and a conserved DNA-binding domain. Thus, it can be inferred that NUPR1 is a highly conserved nuclear protein that binds DNA and undergoes cytoplasmic-to-nuclear translocation [2]. These results are congruent with the previously postulated functions of NUPR1 and provide a sequence context for further studies of its motifs. Previous biophysical work has suggested the presence of posttranslational modification sites that modulate NUPR1 function [6]. Here, we report several likely candidates for posttranslational modification sites, which were identified using extensive bioinformatics analyses and statistical scoring. These sites are amenable to phosphorylation, acetylation, and methylation. However, ubiquitination and sumoylation sites were predicted with low specificity potential. Notably, some of these modification sites fall within regions containing functional linear motifs of NUPR1, making these potential sites of further research interest.

The current study also increases our knowledge of the biophysical properties of NUPR1. We built tridimensional models for NUPR1a, NUPR1b, NUPR2, and the NUPR-like domain of GTF2-I. These models were tested using a number of structural validation methods and rigorous manual scoring. The model of NUPR1a we developed suggests that this protein has a tendency to form a helix-loop-helix motif that is characteristic of other related proteins such as the HMG family of chromatin proteins and transcriptional regulators as well as AT-hooks, which define the HMG-I/Y-subfamily among these proteins. According to official nomenclature, High Mobility Group (HMG) proteins are further classified into three subfamilies: the HMGB (formerly HMG-1/-2) family, the HMGN (formerly HMG-14/-17) family, and the HMGA (formerly HMG-I/Y/C) family [78]. These HMG subfamilies are characterized by the presence of a distinct functional sequence motif. HMGB proteins, for instance, possess a motif known as the “HMG-box,” while the HMGN subfamily contains a “nucleosomal binding domain,” and the HMGA subfamily carries an “AT-hook.” These characteristic functional motifs are widespread among nuclear proteins in a variety of organisms. Consequently, it is accepted that proteins containing any of these functional motifs embedded in their sequence should be known as “HMG motif proteins.” Interestingly, several of these related proteins have a tendency to fold as a helix-loop-helix domain, while many of them—though not all—have a dynamic propensity to disorder (Figs. 4 and 5). These results and models are congruent with data from previous structural studies suggesting that the secondary structure of NUPR1 may be similar to helix-loop-helix motif proteins such as HMG-I/Y (PDB: 1AAB) [79], which also displays a large degree of disorder when isolated in solution [5, 6, 10]. Furthermore, we infer from these models that the 18-amino-acid insertion in NUPR1a adopts the form of a flexible loop. This provides a structural basis for differentiating the two isoforms of NUPR1 for further studies. However, we must also consider that, although the dynamics of many HMG proteins—in particular HMG-I/Y-like proteins—sometimes serve as a barrier to the determination of the structures of their folds, they are still structured as suggested by circular dichroism (CD) and NMR experiments [75]. Many HMG proteins, in particular those outside the HMG-I/Y subfamily, maintain a more robust hydrophobic/aromatic core of the three-helix fold, which is present albeit less pronounced in NUPR1-like family members. These features can be more readily observed in relevant PDB structures such as 2yul, 1i11, 1wz6, 2le4, 2e6o, and 2crj. Thus, it is likely that NUPR1 proteins conserve DNA contacts through a combination of contributions arising from charges and folding. Finally, we underscore the fact that the current work did not explore the contribution of DNA bending to the formation of protein–nucleic acid complexes. Many HMG proteins bind to bent DNA, and the bend angle is often specific to a particular protein. Thus, it is likely that NUPR1-like proteins also share these properties, though careful empirical studies are necessary to support the validity of this idea.

NUPR1 has been implicated in cancer-associated processes, although it remains poorly understood at the mechanistic level [5]. To explore this, we used homology-based methods and docking to develop the first three-dimensional model of NUPR1a bound to DNA. Analyses of this model demonstrate that it could bind to the minor groove of DNA through an HMG-like AT-hook domain, which is part of a loop region. Interface analysis suggests that this complex is maintained through ionic and hydrogen-bonding interactions and reinforced by a second series of basic residues present in the C-terminal domain of the protein. MD simulations reveal that this NUPR1 remains bound to DNA even when undergoing rapid order-to-disorder transitions. Collectively, these results suggest that NUPR1 has the ability to bind to DNA, a fact that has been shown both in vitro and in cultured cells. However, EMSA and biophysical methods have shown that, like several HMGs, NUPR1 has a low affinity and poor sequence specificity for DNA binding [6, 10]. In addition, while these proteins have a propensity to disorder, biophysical methods have also shown that intermolecular interactions stabilize some regions of its sequence. These data do not, however, imply a “conformational selection” scheme [58] for NUPR1–DNA binding, since the HMG-I/Y-like homology-based and DP-Dock modeling approaches used here are ultimately derived using parameters based on single low-energy structures that were experimentally solved. Binding to proteins that have a high degree of disorder is usually explained by two models: folding after binding (also known as “fly casting”) and conformational selection [80]. The first model implies the presence of an intermediate species that shows weak, nonspecific binding, which is followed by folding and specific binding to the target. The second model involves the binding of a ligand to one of the well-folded conformations of the protein. Thus, based on these considerations, it remains possible that other types of NUPR–DNA complexes can be formed depending on the structure and sequence of its target nucleic acid. Lastly, like other transcriptional regulators, NUPR1 forms complexes with other proteins, which could modulate its affinity towards other partners. Posttranslational modifications such as those predicted here and validated experimentally [81] may further modulate the affinity and specificity of this protein for DNA. Therefore, we are optimistic that future studies in which complexes with emerging NUPR1 partners are characterized in detail may help to shed additional light on some important biochemical functions of this protein.

In conclusion, our results strongly suggest that NUPR1 defines a new family of DNA-binding proteins that are related to, yet distinct from, the HMG-I/Y-like subfamily of HMG proteins. Dynamic experiments demonstrate that these proteins are also characterized by their ability to undergo significant order-to-disorder transitions. The intrinsic flexibility of NUPR1 appears to be stabilized by binding to DNA. Furthermore, we report that NUPR1 contains distinct linear motifs which were previously found to mediate nuclear import, export, and DNA binding. Several posttranslational modifications are observed adjacent to or within these motifs. Some of these motifs are modified in vivo (e.g., by PKA and p300) [82]. Consequently, the information reported here should be taken into consideration when designing cell and molecular experiments, as well as during the development of small drugs that can modulate the function of NUPR1-like proteins.

## Electronic supplementary material

Below is the link to the electronic supplementary material.

Table S1 Comparison of sequence identity among NUPR1-like proteins: identity matrix of the multiple sequence alignment in Fig. 1c. The percent identities among the sequences in the alignment are shown. (PDF 710 kb)

Table S2 Intramolecular interactions contributing to the structural integrity of human NUPR1 proteins. **a** Results from calculating the intramolecular interactions reveal that these proteins maintain distinct hydrophobic interaction patterns. Interactions between Leu32 and Leu88 were predicted with this method, further suggesting the existence of conserved interactions among the human NUPR1 proteins. **b–c** Intramolecular salt bridges and hydrogen bonds were predicted for all human NUPR1 proteins, with a maximum salt-bridge distance of 5.0 Å and maximum hydrogen-bond distance of 2.5 Å. These data reveal that these proteins also maintain distinct salt-bridge and hydrogen-bonding patterns. This is congruent with our hypothesis that these proteins share similarities in several biophysical properties but they also maintain distinct interactions that contribute to their structural integrity, in terms of their folding and dynamic conformational changes. (PDF 3349 kb)

Table S3 Assignment of potential posttranslational modification sites within NUPR1a by linear motif analyses. **a** Results of the linear motif analysis for phosphorylation were compiled and statistically scored to assign specificity potential to sites that are predicted to be modified in NUPR1a. For each distinct program, we considered sites for which the prediction score is above the cutoff that had been derived using a training set of modified sequences that were validated experimentally. Subsequently, we developed a meta-prediction score that assigned a maximum score of 1 to sites that were predicted by all of the programs cited. The scores for the other programs were normalized to the maximum score of 1. **b** Acetylation. **c** Methylation. **d** Ubiquitination. **e** Sumoylation. (PDF 1326 kb)

Fig. S1 Potential models and structural templates for NUPR1a. **a** Potential models for NUPR1a were generated using MUSTER [17], I-TASSER [18], QUARK [19], Chunk-TASSER [20], and Pro-sp3-TASSER [21]. Topology diagrams outline the structural comparisons between the generated models. Additionally, a topology diagram for Phf19 (PDB code: 4BD3) is shown. **b** Top-ranked templates used for I-TASSER [18]. *Ident1* refers to the percent identity of the template to the threading-aligned region of the query sequence. *Ident2* refers to the sequence identity of the whole template to that of the query sequence. *Coverage* refers to the number of aligned residues divided by the length of the query protein. Finally, the normalized Z-score is represented for each template. (GIF 52 kb)

High-resolution image (TIFF 23229 kb)
