# Supplementary material for: Evidence supporting the existence of a NUPR1-like family of helix-loop-helix chromatin proteins related to, yet distinct from, AT hook-containing HMG proteins
Source: J Mol Model. 2014 Jul 24;20(8):2357. doi: 10.1007/s00894-014-2357-7 (PMC4139591; doi:10.1007/s00894-014-2357-7)
Supplement: Supplementary file 1 — Comparison of sequence identity among NUPR1-like proteins: identity matrix of the multiple sequence alignment in Fig. 1c. The percent identities among the sequences in the alignment are shown. (PDF 710 kb) [file 894_2014_2357_MOESM1_ESM.pdf]

Supplemental Table 1

1.

Sequence Name	Nupr2 C. porcellus	Nupr2 R. norvegicus	GTF2I DBD	Nupr2 M. musculus	Nupr2 H. sapiens	Nupr1 Drosophila	Nupr1 H. magnipapilla ta	Nupr1 Zebrafish	Nupr1 X. laevis	Nupr1a H. sapiens	Nupr1b H. sapiens	Nupr1 C. porcellus	Nupr1 R. norvegicus	Unnamed M. musculus
**Nupr2 C. porcellus**	100	69.6	51.8	75	73.2	31.6	28.6	46.4	39.3	42.9	42.9	44.6	44.6	42.9
**Nupr2 R. norvegicus**	69.6	100	57.1	92.9	85.7	28.1	30.4	44.6	33.9	41.1	41.1	41.1	39.3	39.3
**GTF2I DBD**	51.8	57.1	100	58.9	66.1	26.3	19.6	33.9	28.6	32.1	32.1	33.9	32.1	32.1
**Nupr2 M. musculus**	75	92.9	58.9	100	89.3	29.8	32.1	46.4	35.7	44.6	44.6	44.6	44.6	42.9
**Nupr2 H. sapiens**	73.2	85.7	66.1	89.3	100	31.6	33.9	48.2	37.5	48.2	48.2	48.2	48.2	46.4
**Nupr1 Drosophila**	31.6	28.1	26.3	29.8	31.6	100	40.4	42.1	40.4	36.8	36.8	42.1	38.6	38.6
**Nupr1 H. magnipapillata**	28.6	30.4	19.6	32.1	33.9	40.4	100	35.7	48.2	44.6	42.9	46.4	46.4	46.4
**Nupr1 Zebrafish**	46.4	44.6	33.9	46.4	48.2	42.1	35.7	100	57.1	51.8	53.6	58.9	52.7	51.8
**Nupr1 X. laevis**	39.3	33.9	28.6	35.7	37.5	40.4	48.2	57.1	100	55.4	55.4	55.4	53.6	53.6
**Nupr1a H. sapiens**	42.9	41.1	32.1	44.6	48.2	36.8	44.6	51.8	55.4	100	91.1	78.6	80.4	76.8
**Nupr1b H. sapiens**	42.9	41.1	32.1	44.6	48.2	36.8	42.9	53.6	55.4	91.1	100	83.6	76.8	80
**Nupr1 C. porcellus**	44.6	41.1	33.9	44.6	48.2	42.1	46.4	58.9	55.4	78.6	83.6	100	76.8	83.6
**Nupr1 R. norvegicus**	44.6	39.3	32.1	44.6	48.2	38.6	46.4	52.7	53.6	80.4	76.8	76.8	100	87.5
**Unnamed M. musculus**	42.9	39.3	32.1	42.9	46.4	38.6	46.4	51.8	53.6	76.8	80	83.6	87.5	100
