# Supplementary material for: Evidence supporting the existence of a NUPR1-like family of helix-loop-helix chromatin proteins related to, yet distinct from, AT hook-containing HMG proteins
Source: J Mol Model. 2014 Jul 24;20(8):2357. doi: 10.1007/s00894-014-2357-7 (PMC4139591; doi:10.1007/s00894-014-2357-7)
Supplement: Supplementary file 2 — Intramolecular interactions contributing to the structural integrity of human NUPR1 proteins. a Results from calculating the intramolecular interactions reveal that these proteins maintain distinct hydrophobic interaction patterns. Interactions between Leu32 and Leu88 were predicted with this method, further suggesting the existence of conserved interactions among the human NUPR1 proteins. b–c Intramolecular salt bridges and hydrogen bonds were predicted for all human NUPR1 proteins, with a maximum salt-bridge distance of 5.0 Å and maximum hydrogen-bond distance of 2.5 Å. These data reveal that these proteins also maintain distinct salt-bridge and hydrogen-bonding patterns. This is congruent with our hypothesis that these proteins share similarities in several biophysical properties but they also maintain distinct interactions that contribute to their structural integrity, in terms of their folding and dynamic conformational changes. (PDF 3349 kb) [file 894_2014_2357_MOESM2_ESM.pdf]

Supplemental Table 2

A

NUPR1a	
**Hydrophobic Interaction**	**Distance (Å)**
A:ALA2 - A:PRO45	4.24042
A:PRO5 - A:MET44	5.0092
A:ALA10 - A:ALA52	3.82275
A:LEU32 - A:LEU88	4.93367
A:ALA33 - A:LEU53	3.23973
A:LEU40 - A:MET42	5.1466
A:MET42 - A:MET44	4.70103
A:PRO43 - A:MET1	4.25479
A:VAL54 - A:LEU29	4.81854
A:ALA98 - A:ARG100	4.57966
A:PHE4 - A:ALA2	4.76838
A:TYR30 - A:ALA33	4.68862
A:TYR30 - A:LEU53	4.66161
A:TYR36 - A:PRO5	4.83244
A:HIS80 - A:PRO77	5.04546

NUPR1b	
**Hydrophobic Interaction**	**Distance (Å)**
:LEU32 - :LEU37	5.09607
:LEU32 - :LEU88	4.19995
:LYS83 - :LYS87	5.12156
:ALA98 - :ARG100	4.68592
:PHE4 - :ALA2	4.11247
:HIS80 - :PRO77	5.09055

NUPR2	
**Hydrophobic Interaction**	**Distance (Å)**
:GLY6+A25+E2:F19+E2:F+E2:F21	3.67522
:CYS43:C,O;ALA45:N - :TYR29	4.49057
:ALA16 - :PRO19	3.57767
:ARG17 - :ILE22	4.59634
:PRO19 - :PRO21	5.30863
:PRO19 - :LYS49	4.61264
:PRO21 - :LYS49	4.5921
:LEU32 - :LEU76	5.37471
:PRO41 - :MET1	4.76504
:CYS43 - :MET1	5.05016
:CYS43 - :LEU12	5.07834
:ALA45 - :LEU12	4.34989
:ARG47 - :ARG59	3.45872
:ARG51 - :LYS49	4.37751
:TYR29 - :LEU32	5.33131
:TYR29 - :CYS43	5.09261
:TYR34 - :ARG7	5.01464
:TYR35 - :LEU37	4.67571
:TYR36 - :ALA5	4.80667
:PHE40 - :ALA3	3.99274
:HIS68 - :LEU37	5.40706

GTF2-I	
**Hydrophobic Interaction**	**Distance (Å)**
A:LEU20:CB - A:HIS18	3.19659
A:ARG5 - A:PRO7	4.88581
A:PRO9 - A:VAL33	3.92254
A:VAL10 - A:LEU16	4.48555
A:LYS13 - A:LYS37	4.72518
A:CYS21 - A:LEU16	4.72855
A:CYS31 - A:LEU46	3.78793
A:ARG35 - A:LEU46	4.26657
A:ALA45 - A:LEU41	5.23032
A:VAL60 - A:LEU41	5.20365
A:TYR22 - A:LEU64	5.11402
A:HIS68 - A:CYS19	5.22373

B

NUPR1a		
**Residue 1**	**Residue 2**	**Distance**
NZ LYS A 95	OD1 ASP A 28	4.94

NUPR1b		
**Residue 1**	**Residue 2**	**Distance**
NH2 ARG 66 7.004	OE1 GLU 67 -3.022	4.27
NH1 ARG 74 -10.491	OE1 GLU 81 -3.217	2.33
NH2 ARG 74 -10.447	OE1 GLU 81 -3.217	3.78
NZ LYS 83 8.013	OE1 GLU 81 -3.217	4.37
NH1 ARG 93 13.477	OD1 ASP 28 -3.214	4.7
NH1 ARG 93 13.477	OD2 ASP 28 -2.607	3.97
NH1 ARG 93 13.477	OE1 GLU 92 1.324	3.44
NH2 ARG 93 14.453	OD1 ASP 25 -5.789	4.82
NH2 ARG 93 14.453	OE1 GLU 26 -12.528	4
NH2 ARG 93 14.453	OD1 ASP 28 -3.214	3.62
NH2 ARG 93 14.453	OD2 ASP 28 -2.607	2.09
NH2 ARG 93 14.453	OE1 GLU 92 1.324	2.09
NZ LYS 95 -0.734	OE1 GLU 26 -12.528	2.57
NZ LYS 95 -0.734	OD1 ASP 28 -3.214	1.67
NZ LYS 95 -0.734	OD2 ASP 28 -2.607	3.67
NZ LYS 95 -0.734	OE1 GLU 92 1.324	3.2
NH1 ARG 96 10.003	OE1 GLU 20 -2.595	2.64
NH1 ARG 96 10.003	OD1 ASP 21 -3.040	2.45
NH1 ARG 96 10.003	OD2 ASP 21 -3.576	4.15
NH1 ARG 96 10.003	OD1 ASP 25 -5.789	3.59
NH1 ARG 96 10.003	OD2 ASP 25 -7.572	3.72
NH2 ARG 96 10.418	OE1 GLU 20 -2.595	1.89
NH2 ARG 96 10.418	OD1 ASP 21 -3.040	4.14
NH2 ARG 96 10.418	OE1 GLU 26 -12.528	4.97
NH1 ARG 99 5.009	OE1 GLU 20 -2.595	1.16
NH1 ARG 99 5.009	OD1 ASP 21 -3.040	4.11
NH2 ARG 99 6.589	OE1 GLU 20 -2.595	0.61
NH2 ARG 99 6.589	OD1 ASP 21 -3.040	2.78
NH2 ARG 99 6.589	OD2 ASP 21 -3.576	4.93

GTF2-I		
**Residue 1**	**Residue 2**	**Distance**
NH1 ARG A 25	OE1 GLU A 14	3.96

NUPR2		
**Residue 1**	**Residue 2**	**Distance**
NH1 ARG 7 -14.702	OD1 ASP 30 -9.898	4.67
NH1 ARG 7 -14.702	OD2 ASP 30 -10.215	4.66
NH1 ARG 11 -17.709	OE1 GLU 26 -12.654	2.86
NH1 ARG 11 -17.709	OE1 GLU 27 -4.640	2.96
NH2 ARG 11 -15.770	OE1 GLU 26 -12.654	2.8
NH2 ARG 11 -15.770	OE1 GLU 27 -4.640	3.72
NH1 ARG 38 -5.249	OD1 ASP 39 -6.924	3.85
NH2 ARG 38 -3.168	OD1 ASP 39 -6.924	2.88
NH2 ARG 38 -3.168	OD2 ASP 39 -6.114	4.6
NH1 ARG 47 -8.372	OE1 GLU 55 -0.384	3.35
NH2 ARG 47 -8.701	OE1 GLU 55 -0.384	1.83
NH1 ARG 53 2.744	OE1 GLU 25 -8.657	2.12
NH1 ARG 53 2.744	OE1 GLU 26 -12.654	4.69
NH2 ARG 53 4.264	OE1 GLU 25 -8.657	2.13
NH2 ARG 53 4.264	OE1 GLU 26 -12.654	4.89
NH1 ARG 59 -5.330	OE1 GLU 55 -0.384	3.24
NH2 ARG 59 -3.425	OE1 GLU 55 -0.384	3.38
ND1 HIS 68 -4.514	OE1 GLU 2 -20.368	4.66
ND1 HIS 68 -4.514	OD1 ASP 39 -6.924	4.11
ND1 HIS 68 -4.514	OD2 ASP 39 -6.114	2.91
NE2 HIS 68 -2.877	OD1 ASP 39 -6.924	2.68
NE2 HIS 68 -2.877	OD2 ASP 39 -6.114	2.08
NH1 ARG 70 5.231	OE1 GLU 69 -4.156	4.85
NZ LYS 71 3.420	OE1 GLU 2 -20.368	4.61
NZ LYS 71 3.420	OD1 ASP 39 -6.924	3.92
NZ LYS 71 3.420	OD2 ASP 39 -6.114	2.85
NZ LYS 75 4.049	OE1 GLU 2 -20.368	1.42
NH1 ARG 81 11.719	OE1 GLU 26 -12.654	1.04
NH2 ARG 81 12.925	OE1 GLU 26 -12.654	2.1
NH2 ARG 81 12.925	OE1 GLU 27 -4.640	4.39
NZ LYS 82 11.705	OD2 ASP 30 -10.215	3.45
NH1 ARG 83 2.630	OD1 ASP 30 -9.898	1.23
NH1 ARG 83 2.630	OD2 ASP 30 -10.215	2.33
NH1 ARG 83 2.630	OD2 ASP 33 -12.111	3.81
NH2 ARG 83 2.067	OD1 ASP 30 -9.898	1.08
NH2 ARG 83 2.067	OD2 ASP 30 -10.215	2.4
NH2 ARG 83 2.067	OD2 ASP 33 -12.111	4.23
NH1 ARG 84 8.358	OE1 GLU 27 -4.640	4.16

C

NUPR1a		NUPR1b		NUPR2		GTF2-I	
**Hydrogen Bond Interaction**	**Distance (Å)**	**Hydrogen Bond Interaction**	**Distance (Å)**	**Hydrogen Bond Interaction**	**Distance (Å)**	**Hydrogen Bond Interaction**	**Distance (Å)**
A:GLN12:N - A:THR55:OG1	3.03931	:LYS95:NZ - :ASP28:OD1	2.99083	:ARG84:NH1 - :LEU88:OXT	3.12783	A:ARG25:NH1 - A:GLU14:OE2	2.88534
A:SER22:N - A:ASP19:O	2.755	:GLU18:N - :GLU18:OE1	2.904	:ARG7:NH1 - :ARG7:O	3.16373	A:GLN1:NE2 - A:ALA2:O	3.1485
A:SER23:N - A:GLU20:O	3.09114	:SER22:N - :ASP19:O	2.66519	:ARG11:NE - :GLU26:OE2	3.1962	A:SER11:OG - A:SER36:OG	2.69727
A:SER23:OG - A:GLU20:O	2.57438	:SER23:N - :GLU20:O	3.13578	:ARG17:N - :LEU15:O	2.60325	A:GLU15:N - A:TYR22:OH	2.85154
A:LEU24:N - A:GLU20:O	2.9626	:SER23:OG - :GLU20:O	2.61153	:ILE22:N - :PRO19:O	2.61896	A:SER17:OG - A:GLU15:O	2.90501
A:LEU24:N - A:ASP21:O	3.20057	:LEU24:N - :GLU20:O	2.93899	:SER23:N - :PRO20:O	3.16265	A:CYS21:N - A:HIS18:O	2.84939
A:ASP25:N - A:ASP21:O	2.82127	:LEU24:N - :ASP21:O	3.21552	:SER23:OG - :PRO20:O	2.57748	A:TYR22:N - A:HIS18:O	2.9294
A:ASP25:N - A:SER22:O	3.2982	:ASP25:N - :ASP21:O	2.61665	:TYR24:N - :PRO20:O	3.00399	A:TYR22:N - A:CYS19:O	3.0758
A:GLU26:N - A:SER22:O	3.13418	:ASP25:N - :SER22:O	3.11485	:TYR24:N - :PRO21:O	3.2463	A:TYR23:N - A:CYS19:O	2.85259
A:GLU26:N - A:SER23:O	3.25681	:GLU26:N - :SER22:O	3.05907	:GLU25:N - :PRO21:O	2.61214	A:TYR23:N - A:LEU20:O	2.98498
A:SER27:N - A:SER23:O	2.96121	:GLU26:N - :SER23:O	3.10352	:GLU25:N - :ILE22:O	3.10433	A:TYR24:N - A:LEU20:O	2.73441
A:SER27:N - A:LEU24:O	3.23815	:SER27:N - :SER23:O	3.07079	:GLU26:N - :ILE22:O	2.84691	A:TYR24:N - A:CYS21:O	3.37641
A:SER27:OG - A:SER23:O	3.22569	:SER27:N - :LEU24:O	3.30242	:GLU26:N - :SER23:O	3.07109	A:ARG25:N - A:CYS21:O	3.06278
A:ASP28:N - A:LEU24:O	2.84588	:ASP28:N - :LEU24:O	2.85004	:GLU27:N - :SER23:O	3.00624	A:ARG25:N - A:TYR22:O	3.17086
A:ASP28:N - A:ASP25:O	3.12206	:ASP28:N - :ASP25:O	3.23326	:GLU27:N - :TYR24:O	3.35049	A:ARG26:N - A:TYR22:O	2.9753
A:LEU29:N - A:ASP25:O	2.82039	:LEU29:N - :ASP25:O	2.6138	:LEU28:N - :TYR24:O	2.74252	A:ARG26:N - A:TYR23:O	3.21074
A:LEU29:N - A:GLU26:O	3.36728	:LEU29:N - :GLU26:O	3.21928	:LEU28:N - :GLU25:O	3.36066	A:ARG26:NH1 - A:LYS57:O	2.7765
A:TYR30:N - A:GLU26:O	2.58894	:TYR30:N - :GLU26:O	2.5971	:TYR29:N - :GLU25:O	2.59368	A:ASP27:N - A:TYR23:O	3.04512
A:SER31:N - A:SER27:O	2.58955	:TYR30:N - :SER27:O	3.14954	:TYR29:N - :GLU26:O	3.29774	A:ASP27:N - A:TYR24:O	3.3071
A:LEU32:N - A:LEU29:O	3.12251	:SER31:N - :SER27:O	2.70531	:ASP30:N - :GLU26:O	2.60135	A:CYS31:SG - A:LEU46:O	2.85478
A:ALA33:N - A:LEU29:O	3.02086	:LEU32:N - :ASP28:O	2.9997	:ASP30:N - :GLU27:O	3.14044	A:ARG35:NE - A:GLU43:O	3.04981
A:ALA33:N - A:TYR30:O	2.93116	:ALA33:N - :LEU29:O	2.59174	:CYS31:N - :GLU27:O	3.05109	A:LYS37:N - A:TYR12:O	3.21818
A:HIS34:N - A:TYR30:O	2.92395	:ALA33:N - :TYR30:O	2.70753	:LEU32:N - :LEU28:O	2.86233	A:SER42:N - A:LEU39:O	2.76896
A:SER35:OG - A:LEU32:O	3.19378	:HIS34:N - :SER31:O	3.36163	:LEU32:N - :TYR29:O	3.22751	A:GLU43:N - A:LEU39:O	2.85
A:MET44:N - A:ALA2:O	2.99543	:TYR36:OH - :THR3:O	2.81888	:ASP33:N - :TYR29:O	2.57938	A:GLU43:N - A:THR40:O	3.11041
A:ALA52:N - A:THR50:OG1	3.35566	:LYS61:N - :GLY59:O	2.61764	:ASP33:N - :ASP30:O	2.77278	A:GLN44:N - A:THR40:O	3.00321
A:LEU53:N - A:THR8:O	3.04413	:ARG63:N - :ARG60:O	3.22492	:TYR34:N - :ASP30:O	3.03536	A:GLN44:N - A:LEU41:O	3.23663
A:THR55:OG1 - A:ALA10:O	2.57162	:ARG63:NE - :ARG60:O	3.28211	:TYR34:N - :CYS31:O	3.06081	A:ALA45:N - A:LEU41:O	2.90526
A:ARG60:NH2 - A:ARG63:O	3.25563	:GLU67:N - :THR64:OG1	3.25027	:TYR34:OH - :ARG7:O	3.12179	A:ALA45:N - A:SER42:O	3.26038
A:GLU67:N - A:THR64:OG1	2.89665	:GLU67:N - :THR64:O	3.01846	:ARG53:NH1 - :GLN56:OE1	2.96899	A:LEU46:N - A:SER42:O	2.92362
A:GLU67:N - A:THR64:O	2.88702	:ALA68:N - :THR64:O	2.93231	:GLU55:N - :THR52:O	3.19619	A:LEU46:N - A:GLU43:O	3.17426
A:ALA68:N - A:THR64:O	2.94409	:ALA68:N - :LYS65:O	3.1327	:GLN56:N - :THR52:O	2.68566	A:HIS47:N - A:GLU43:O	2.79786
A:ALA68:N - A:LYS65:O	3.23318	:ALA69:N - :LYS65:O	2.86889	:GLN56:N - :ARG53:O	2.81945	A:HIS47:N - A:GLN44:O	3.0115
A:ALA69:N - A:LYS65:O	2.87997	:ALA69:N - :ARG66:O	3.16876	:ALA57:N - :ARG53:O	2.88355	A:THR48:N - A:GLN44:O	2.79802
A:ALA69:N - A:ARG66:O	3.22316	:ALA70:N - :ARG66:O	2.96919	:ALA57:N - :ARG54:O	2.91061	A:LYS57:NZ - A:ARG50:O	3.09601
A:ALA70:N - A:ARG66:O	2.89458	:ALA70:N - :GLU67:O	3.10751	:LEU58:N - :ARG54:O	3.13806	A:GLN58:N - A:GLY55:O	2.81228
A:ALA70:N - A:GLU67:O	3.16008	:ASN71:N - :GLU67:O	3.03869	:LEU58:N - :GLU55:O	2.97948	A:LYS59:N - A:GLY55:O	2.76433
A:ASN71:N - A:GLU67:O	2.9865	:ASN71:N - :ALA68:O	3.1353	:ARG59:N - :GLU55:O	2.80996	A:LYS59:N - A:HIS56:O	3.09886
A:ASN71:N - A:ALA68:O	3.12963	:THR72:N - :ALA68:O	2.74547	:ARG59:N - :GLN56:O	3.10134	A:VAL60:N - A:HIS56:O	2.99195
A:THR72:N - A:ALA68:O	2.9302	:THR72:N - :ALA69:O	3.09381	:THR60:N - :GLN56:O	2.80058	A:VAL60:N - A:LYS57:O	3.22274
A:THR72:N - A:ALA69:O	3.15449	:THR72:OG1 - :ALA68:O	2.59837	:THR60:N - :ALA57:O	3.22261	A:GLY61:N - A:LYS57:O	2.99782
A:THR72:OG1 - A:ALA68:O	2.86529	:ASN73:N - :ALA69:O	2.64923	:THR60:OG1 - :GLN56:O	2.90212	A:GLY61:N - A:GLN58:O	3.15478
A:ASN73:N - A:ALA69:O	2.7656	:ASN73:N - :ALA70:O	3.04184	:ASN61:N - :ALA57:O	2.69489	A:GLN62:N - A:GLN58:O	2.88262
A:ASN73:N - A:ALA70:O	3.00822	:ARG74:N - :GLU81:OE2	3.21664	:ASN61:N - :LEU58:O	3.00772	A:GLN62:N - A:LYS59:O	3.21943
A:ASN73:ND2 - A:ALA69:O	2.70977	:ARG74:NH2 - :MET1:O	2.94675	:TRP62:N - :THR60:O	2.74931	A:LYS63:N - A:LYS59:O	2.89719
A:ARG74:N - A:GLU81:OE2	3.29468	:GLY79:N - :SER76:O	2.74648	:GLY67:N - :ALA64:O	2.73987	A:LYS63:N - A:VAL60:O	3.09803
A:ARG74:NE - A:THR46:O	2.66147	:ARG82:N - :GLY79:O	2.86375	:ARG70:N - :GLY67:O	3.05563	A:LEU64:N - A:VAL60:O	2.8133
A:GLY79:N - A:SER76:O	2.99764	:LYS83:N - :GLY79:O	2.9015	:LYS71:N - :GLY67:O	3.1254	A:LEU64:N - A:GLY61:O	3.20017
A:ARG82:N - A:GLY79:O	2.66076	:LYS83:N - :HIS80:O	3.04346	:VAL72:N - :HIS68:O	2.61868	A:LEU65:N - A:GLY61:O	2.92778
A:LYS83:N - A:GLY79:O	2.87315	:LEU84:N - :HIS80:O	2.76363	:VAL72:N - :GLU69:O	3.3231	A:LEU65:N - A:GLN62:O	3.14774
A:LYS83:N - A:HIS80:O	3.19759	:LEU84:N - :GLU81:O	3.07496	:ALA73:N - :GLU69:O	2.95864	A:ASN66:N - A:GLN62:O	2.93845
A:LEU84:N - A:HIS80:O	2.82042	:VAL85:N - :GLU81:O	2.96131	:ALA73:N - :ARG70:O	3.18302	A:ASN66:N - A:LYS63:O	3.23633
A:LEU84:N - A:GLU81:O	3.17224	:VAL85:N - :ARG82:O	3.09545	:GLN74:N - :ARG70:O	3.16815	A:ASN66:ND2 - A:GLN62:OE1	3.05877
A:VAL85:N - A:GLU81:O	2.93547	:THR86:N - :ARG82:O	2.88599	:GLN74:N - :LYS71:O	3.08889	A:ASN66:ND2 - A:GLN62:O	2.84412
A:VAL85:N - A:ARG82:O	3.14906	:THR86:N - :LYS83:O	3.09737	:LYS75:N - :LYS71:O	3.17582	A:HIS68:N - A:LEU64:O	2.94295
A:THR86:N - A:ARG82:O	2.91848	:THR86:OG1 - :LYS83:O	2.57735	:LYS75:N - :VAL72:O	3.23719	A:HIS68:N - A:LEU65:O	3.1177
A:THR86:N - A:LYS83:O	3.09465	:LYS87:N - :LYS83:O	2.90752	:LYS75:NZ - :LYS71:O	3.31156	A:CYS69:N - A:LEU65:O	3.04173
A:THR86:OG1 - A:LYS83:O	2.57868	:LYS87:N - :LEU84:O	3.09657	:LEU76:N - :VAL72:O	2.83717	A:CYS69:SG - A:LEU65:O	3.07248
A:LYS87:N - A:LYS83:O	2.92525	:LEU88:N - :LEU84:O	2.84891	:LEU76:N - :ALA73:O	3.127	A:PRO7:CD - A:ARG5:O	2.79442
A:LYS87:N - A:LEU84:O	3.20894	:LEU88:N - :VAL85:O	3.00255	:LEU77:N - :ALA73:O	2.74628	A:LYS13:CA - A:LYS37:O	2.72414
A:LEU88:N - A:LEU84:O	2.97109	:GLN89:N - :VAL85:O	2.88406	:LEU77:N - :GLN74:O	2.95329	A:ARG50:CD - A:ARG26:O	3.4997
A:LEU88:N - A:VAL85:O	3.13787	:GLN89:N - :THR86:O	3.09478	:ASN78:N - :GLN74:O	3.17967	A:ARG5:N - A:TYR24	3.76512
A:GLN89:N - A:VAL85:O	2.89728	:ASN90:N - :THR86:O	2.84944	:ASN78:N - :LYS75:O	3.01816	A:TYR24:OH - A:PHE28	3.83195
A:GLN89:N - A:THR86:O	3.1583	:ASN90:N - :LYS87:O	3.16852	:GLY79:N - :LYS75:O	3.32515		
A:ASN90:N - A:THR86:O	2.89899	:SER91:N - :LYS87:O	2.88344	:GLY79:N - :LEU76:O	3.13317		
A:ASN90:N - A:LYS87:O	3.16425	:SER91:N - :LEU88:O	3.06893	:GLN80:N - :LEU76:O	2.80169		
A:SER91:N - A:LYS87:O	3.02698	:SER91:OG - :LYS87:O	3.12736	:GLN80:N - :LEU77:O	3.15636		
A:SER91:N - A:LEU88:O	3.17122	:GLU92:N - :LEU88:O	3.01396	:ARG81:N - :LEU77:O	2.70909		
A:SER91:OG - A:LYS87:O	3.38476	:GLU92:N - :GLN89:O	2.94388	:ARG81:N - :ASN78:O	3.16007		
A:GLU92:N - A:LEU88:O	3.0318	:ARG93:N - :GLN89:O	2.9153	:LYS82:N - :GLY79:O	3.13685		
A:GLU92:N - A:GLN89:O	3.13366	:ARG93:N - :ASN90:O	2.91536	:ARG83:N - :GLY79:O	3.10776		
A:ARG93:N - A:GLN89:O	2.87614	:LYS94:N - :ASN90:O	2.91879	:ARG83:N - :GLN80:O	2.95613		
A:ARG93:N - A:ASN90:O	3.05366	:LYS94:N - :SER91:O	3.21564	:ARG84:N - :GLN80:O	2.60223		
A:LYS94:N - A:ASN90:O	2.96325	:LYS95:N - :SER91:O	2.90722	:ARG84:N - :ARG81:O	2.93641		
A:LYS94:N - A:SER91:O	3.166	:LYS95:N - :GLU92:O	3.12861	:ARG84:NH1 - :ARG84:O	2.92188		
A:LYS95:N - A:SER91:O	3.00018	:ARG96:N - :GLU92:O	2.82389	:GLN85:N - :ARG81:O	2.95191		
A:LYS95:N - A:GLU92:O	3.06876	:ARG96:N - :ARG93:O	3.0914	:GLN85:N - :LYS82:O	3.03396		
A:ARG96:N - A:GLU92:O	2.80282	:GLY97:N - :ARG93:O	2.92032	:ARG86:N - :LYS82:O	3.27791		
A:ARG96:N - A:ARG93:O	3.12843	:GLY97:N - :LYS94:O	3.02998	:ARG86:N - :ARG83:O	3.20722		
A:ARG96:NH1 - A:ARG96:O	2.82302	:ALA98:N - :LYS94:O	2.82891	:GLN87:N - :ARG83:O	2.60479		
A:GLY97:N - A:ARG93:O	2.91127	:ALA98:N - :LYS95:O	3.30455	:GLN87:N - :ARG84:O	2.9807		
A:GLY97:N - A:LYS94:O	3.1068	:ARG99:N - :LYS95:O	2.61092	:PRO10:CD - :ALA8:O	2.94218		
A:ALA98:N - A:LYS94:O	2.84368	:PRO6:CD - :PHE4:O	3.77412	:ILE22:CA - :GLU25:OE1	3.59923		
A:PRO6:CD - A:PHE4:O	3.58026	:SER9:CB - :TYR30:OH	3.40467	:GLY46:CA - :GLU25:OE2	3.79173		
A:PRO11:CD - A:SER9:O	2.8155	:PRO11:CD - :SER9:O	2.9035	:ARG53:CD - :GLN56:OE1	3.33039		
A:SER22:CB - A:GLY16:O	2.74698	:SER22:CB - :PRO17:O	3.23614	:TRP62:CD1 - :PRO63:O	3.46241		
A:SER22:CB - A:PRO17:O	3.27192	:SER35:CB - :LEU32:O	2.72314				
A:SER31:CA - A:HIS34:NE2	3.33118	:LYS95:CE - :GLU92:OE1	3.33839				
A:HIS34:CD2 - A:TYR30:O	2.78667						
A:PRO43:CD - A:ILE41:O	2.82196						
A:PRO51:CD - A:LEU49:O	2.79773						
A:PRO75:CD - A:ASN73:O	3.51577						
