# Supplementary material for: Evidence supporting the existence of a NUPR1-like family of helix-loop-helix chromatin proteins related to, yet distinct from, AT hook-containing HMG proteins
Source: J Mol Model. 2014 Jul 24;20(8):2357. doi: 10.1007/s00894-014-2357-7 (PMC4139591; doi:10.1007/s00894-014-2357-7)
Supplement: Supplementary file 3 — Assignment of potential posttranslational modification sites within NUPR1a by linear motif analyses. a Results of the linear motif analysis for phosphorylation were compiled and statistically scored to assign specificity potential to sites that are predicted to be modified in NUPR1a. For each distinct program, we considered sites for which the prediction score is above the cutoff that had been derived using a training set of modified sequences that were validated experimentally. Subsequently, we developed a meta-prediction score that assigned a maximum score of 1 to sites that were predicted by all of the programs cited. The scores for the other programs were normalized to the maximum score of 1. b Acetylation. c Methylation. d Ubiquitination. e Sumoylation. (PDF 1326 kb) [file 894_2014_2357_MOESM3_ESM.pdf]

Supplemental Table 3

A

Position	NetPhosk 1.0	NetPhos 2.0	Kinasephos 2	DIPHOS	Phospho SVM	Scansite	Musite	PPSP	GPS	Predicted Kinases	Sequence Region	Assigned Score
T-3	+	-	-	+	+	-	-	-	+	PKC	MATFPPATSAPQ	0.44
T-8	-	-	-	-	-	-	-	-	+	CMGC, GSK, CDK, CDK4	MATFPPATSAPQQPP	0.11
S-9	-	-	-	-	-	-	-	-	+	CMGC, GSK, GSK3B, IKK, IKKb	ATFPPATSAPQQPPG	0.11
S-22	+	+	+	+	-	+	+	+	+	CKI, CKII, ATM, GRK	PGPEDEDSSLDESDL	0.88
S-23	+	+	+	+	+	+	+	+	+	CKII, DNAPK, ATM, PLK1	GPEDEDS**S**LDESDLY	1
S-27	+	-	+	+	+	-	+	+	+	CKII, cdc2, ATM, PLK1, MAPKKK	SLDESDLYS	0.77
Y-30	+	-	-	+	+	+	+	-	+	SRC, INSR, Itk Kinase	SLDESDL**Y**SLAHSYL	0.66
S-31	-	-	+	+	+	-	-	+	+	ATM, MAPKKK	SDLYSLAHS	0.55
S-35	+	+	+	+	+	-	-	+	+	cdc2, ATM	SLAHSYLGP	0.77
Y-36	-	-	-	-	+	-	+	-	+	TK, Eph, VEGFR, KDR	LYSLAHSYLGPLIMP	0.33
T-46	-	-	-	-	-	-	-	-	+	CMGC, GSK, GSK3A	PLIMPMPTSPLTPAL	0.11
S-47	+	-	+	+	+	-	+	+	+	PLK, PLK1, PEK, PKR	PMPTSPLTP	0.77
T-50	+	-	+	+	+	-	+	+	+	p38MAPK, GSK3, Erk1 Kinase	PMPTSPL**T**PALVTGG	0.77
T-55	-	-	-	-	+	-	-	-	+	AGC, PKC, Alpha, PKCg	TSPLTPALVTGGGGR KGRT	0.22
T-64	+	+	-	+	+	-	+	-	+	PKC, cdc2	TGGGGRKGRTKREAA ANTN	0.66
T-72	+	+	-	+	-	-	-	-	+	PKC, AGC, DMPK, ROCK	KREAAANTNRPSPGG	0.44
S-76	+	+	+	+	+	+	-	-	+	GSK3, ATM, Aurora, cdk5	AANTNRPSPGGHERK	0.77
T-86	+	-	-	-	+	-	-	-	+	PKC	PGGHERKLVTKLQNS ERKK	0.33
S-91	+	-	+	+	-	-	-	+	+	PKC, ATM, Aurora, MAPKKK	KLQNSERKK	0.55

B

Position	PAIL	PREDMOD	ASEB	PLMLA	PSKAcePred	BRABSB-PHKA	LysAcet	EnsemblePail	Sequence Region	Assigned Score
K-61	+	+	+	+	+	+	+	-	TGGGGR-K-GRTKRE	0.875
K-65	+	+	+	+	-	-	+	-	GRKGRT-K-REAAAN	0.75
K-83	+	+	+	+	-	-	+	+	PGGHER-K-LVTKLQ	0.75
K-87	+	-	-	+	+	-	-	+	ERKLVT-K-LQNSER	0.5
K-94	+	-	-	+	-	-	+	-	LQNSER-K-KRGARR	0.375
K-95	+	+	-	-	-	-	-	+	QNSERK-K-RGARR-	0.375

C

Position	PMeS	BPB-PPMS	PLMLA	CSKAAP MetSite	Sequence Region	Assigned Score
R-60	-	-	-	+	N/A	0.25
K-61	-	+	-	-	GGGGRKGRTKR	0.25
R-63	-	-	-	+	N/A	0.25
K-65	+	-	+	-	GRKGRTKREAAAN	0.5
R-66	-	-	-	+	N/A	0.25
R-74	-	-	-	+	EAAANTNRPSPGGHE	0.25
R-82	-	-	-	+	N/A	0.25
K-94	+	+	+	-	LQNSERKKRGARR	0.75

D

Position	BDB-PUB	CKSAAP UbSite	UbPred	Sequence Region	Assigned Score
K-61	+	-	-	VTGGGGRKGRTKREA	0.33
K-65	+	-	-	GGRKGRTKREAAANT	0.33
K-83	+	-	-	SPGGHERKLVTKLQN	0.33
K-87	+	-	-	HERKLVTKLQNSERK	0.33
K-94	+	-	-	KLQNSERKKRGARR	0.33
K-95	+	-	-	LQNSERKKRGARR	0.33

E

Position	SUMOsp 1.0	SUMOsp 2.0	SUMOplot	SUMOhydro	PCI-SUMO	GPS-SBM 1.0	ELM	Sequence Region	Assigned Score
K-61	-	-	-	-	-	-	-	N/A	0
K-65	-	+	-	-	+	-	-	GGGRKGRTKREAAA NTN	0.33
K-83	-	-	-	-	-	-	-	N/A	0
K-87	-	-	-	-	-	-	-	N/A	0
K-94	-	-	-	-	-	-	-	N/A	0
K-95	-	-	+	-	-	-	-	QNSERKKRGARR	0.1666667
