# Supplementary figures and images for: Evidence supporting the existence of a NUPR1-like family of helix-loop-helix chromatin proteins related to, yet distinct from, AT hook-containing HMG proteins
Source: J Mol Model. 2014 Jul 24;20(8):2357. doi: 10.1007/s00894-014-2357-7 (PMC4139591; doi:10.1007/s00894-014-2357-7)

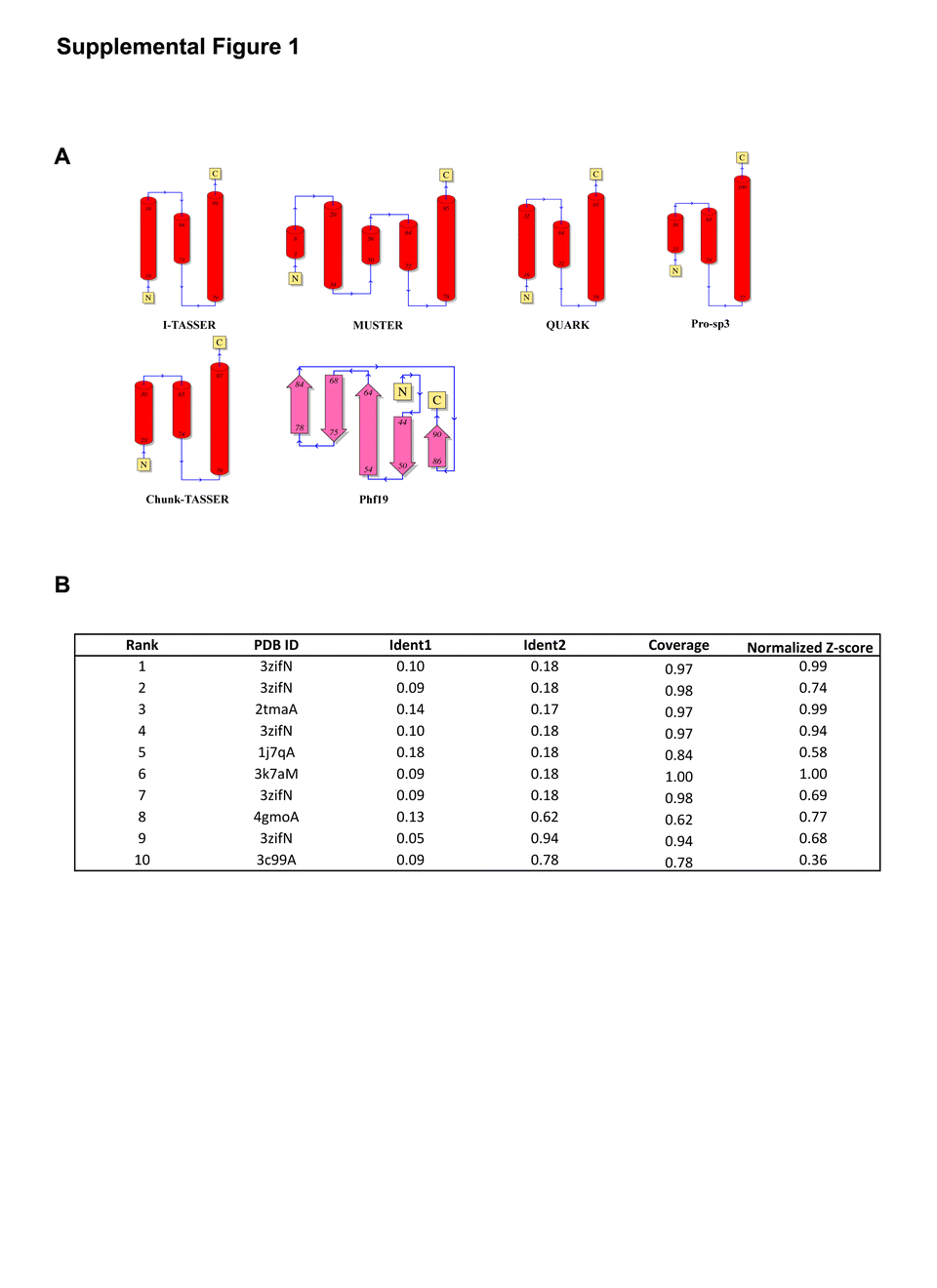

Supplement: Supplementary file 4 — Potential models and structural templates for NUPR1a. a Potential models for NUPR1a were generated using MUSTER [17], I-TASSER [18], QUARK [19], Chunk-TASSER [20], and Pro-sp3-TASSER [21]. Topology diagrams outline the structural comparisons between the generated models. Additionally, a topology diagram for Phf19 (PDB code: 4BD3) is shown. b Top-ranked templates used for I-TASSER [18]. Ident1 refers to the percent identity of the template to the threading-aligned region of the query sequence. Ident2 refers to the sequence identity of the whole template to that of the query sequence. Coverage refers to the number of aligned residues divided by the length of the query protein. Finally, the normalized Z-score is represented for each template. (GIF 52 kb) [file 894_2014_2357_Fig9_ESM.gif]

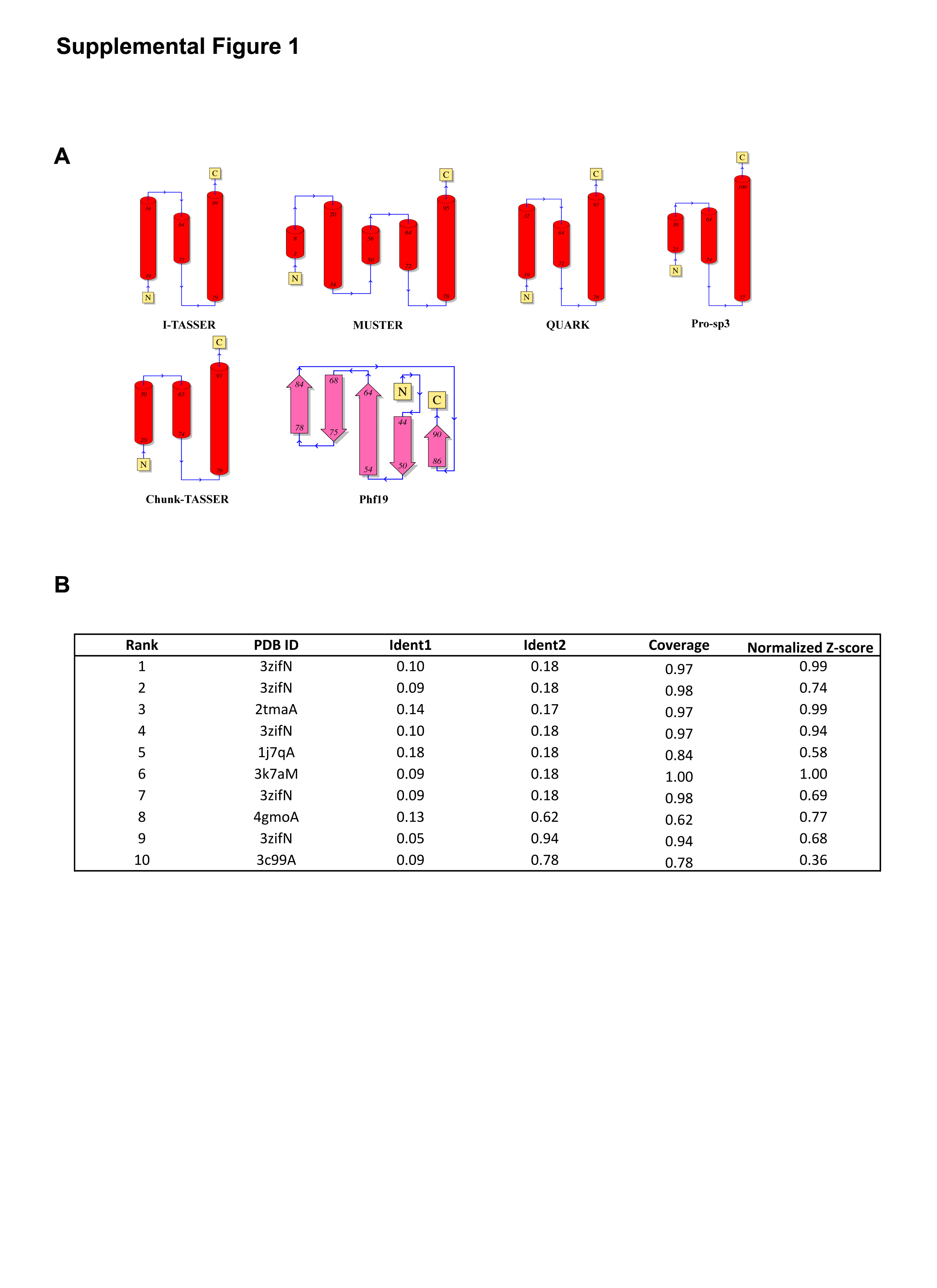

Supplement: Supplementary file 5 — High-resolution image (TIFF 23229 kb) [file 894_2014_2357_MOESM5_ESM.tif]
